# Mathematical modeling of malaria vaccination with seasonality and immune feedback

**DOI:** 10.1371/journal.pcbi.1012988

**Published:** 2025-05-12

**Authors:** Zhuolin Qu, Denis Patterson, Lihong Zhao, Joan Ponce, Christina J. Edholm, Olivia F. Prosper Feldman, Lauren M. Childs

**Affiliations:** 1 Department of Mathematics, University of Texas at San Antonio, San Antonio, Texas, United States of America; 2 Department of Mathematical Sciences, Durham University, Durham, United Kingdom; 3 Department of Applied Mathematics, University of California, Merced, Merced, California, United States of America; 4 Department of Mathematics, Virginia Tech, Blacksburg, Virginia, United States of America; 5 School of Mathematical and Statistical Sciences, Arizona State University, Tempe, Arizona, United States of America; 6 Mathematics Department, Scripps College, Claremont, California, United States of America; 7 Department of Mathematics, University of Tennessee, Knoxville, Tennessee, United States of America; 8 Virginia Tech Center for the Mathematics of Biosystems, Virginia Tech, Blacksburg, Virginia, United States of America; National University of Singapore Public Health, SINGAPORE

## Abstract

Malaria is one of the deadliest infectious diseases globally, claiming hundreds of thousands of lives each year. The disease presents substantial heterogeneity among the population, with approximately two-thirds of fatalities occurring in children under five years old. Immunity to malaria develops through repeated exposure and plays a crucial role in disease dynamics. Seasonal environmental fluctuations, such as changes in temperature and rainfall, lead to temporal heterogeneity and further complicate transmission dynamics and the utility of intervention strategies. We employ an age-structured partial differential equation model to characterize seasonal malaria transmission and assess vaccination strategies that vary by timing and duration. Our model integrates vector-host epidemiological dynamics across different age groups and nonlinear feedback between transmission and immunity. We calibrate the model to year-round and seasonal malaria settings and conduct extensive sensitivity analyses for both scenarios to systematically assess which assumptions lead to the most uncertainty. We use time-varying sensitivity indices to identify critical disease parameters during low and high transmission seasons. We further investigate the impact of vaccination and its implementation in the seasonal malaria settings. When implementing a three-dose primary vaccination series, seasonally targeted campaigns can prevent significantly more cases per vaccination than constant year-long programs in regions with strong seasonal variation in transmission. In such scenarios, the optimal vaccination interval aligns with the peak in infected mosquito abundance and precedes the peak in malaria transmission. In contrast, seasonal booster programs may provide limited advantages over year-long vaccination. Additionally, while increasing annual vaccination counts can reduce overall disease incidence, it yields marginal improvements in cases prevented per vaccination.

## Author summary

Malaria remains a significant health concern worldwide, especially for children under age five, who account for two-thirds of malaria-related deaths. Our work focuses on understanding how malaria spreads and how factors like immunity, seasonal changes, and vaccination strategies affect its spread. We use a mathematical model to simulate malaria transmission between humans and mosquitoes in different regions, including year-round malaria transmission and seasonal outbreaks. We determine the most effective vaccine distribution strategies by studying how malaria rates vary between age groups and seasons. Our results show that targeted vaccination campaigns during specific months are potentially more effective than vaccinating people year-round in utilizing limited vaccinations when malaria is highly seasonal. Such targeted approaches can prevent more cases per vaccination, and the ideal vaccination window aligns with the period when infectious mosquitoes are most abundant, but malaria transmission has yet to peak. These findings highlight the need for careful vaccination planning and tailored strategies to combat malaria effectively.

## 1. Introduction

Malaria, caused by parasites from the *Plasmodium* genus, is one of the most harmful human diseases, causing more than 600,000 deaths and over 249 million cases each year [1]. Globally, more than 50% of the world’s population lives in regions where malaria is endemic. Most of the disease burden is concentrated in sub-Saharan Africa, where the *Plasmodium falciparum* parasite is the most prevalent species [2]. Despite widespread efforts over the past century to eradicate malaria, it remains a significant burden on global public health, and recent evidence suggests that progress in combating the disease has slowed or even stopped in some countries [1, 3, 4]. The mosquito vector has been the target of most interventions, mainly due to a poor understanding of the complex immune response to malaria infection and the lack of an effective and long-lasting malaria vaccine [5, 6]. However, mosquito-targeting interventions are losing efficacy as insecticide resistance increases and spreads [5, 7]. When humans experience regular exposure to malaria parasites, they typically acquire significant protection against symptomatic disease [8], meaning that detailed modeling of immune landscapes is crucial to forecasting malaria dynamics in human populations. Furthermore, protection against malaria differs significantly based on age and location. Thus, understanding the impact of realistic immune feedback is pivotal for effective disease management across various geographic and demographic contexts [9–11].

Malaria transmission is highly heterogeneous, with incidence profiles varying significantly by location [12]. In many regions of sub-Saharan Africa, malaria incidence is highly seasonal and impacted by changes in temperature and rainfall. When malaria transmission is strongly seasonal, the age distribution of infection typically displays a dramatically higher disease burden among older children [13]. Therefore, management strategies must adapt to address the shift in affected ages to maximize protection. For example, in northern Ghana, where seasonality is very pronounced, intermittent preventive treatment for infants (IPTi) was found to be substantially less effective than in areas with milder seasonal effects [14]. This realization resulted in studies on changing intermittent preventive treatment (IPT) implementation for age ranges based on whether or not a region has seasonal transmission [14–19]. Recently, in addition to IPT and traditional management strategies, the implementation of WHO-recommended vaccines to prevent malaria cases is being considered. There are three categories of malaria vaccines, each targeting a different developmental stage of the parasite. Currently, only two malaria vaccines have received WHO recommendation—RTS,S/AS01 in October 2021 and R21/Matrix-M in October 2023 [20], both of which are pre-erythrocytic vaccines that target antigens from *Plasmodium* sporozoites and liver stages [6, 21]. The other two categories of vaccines are blood-stage and transmission-blocking [6, 21], with several candidate vaccines currently undergoing different stages of trials. Both RTS,S/AS01 and R21/Matrix-M are shown to be safe and effective in preventing malaria in children and can reduce about 75% of symptomatic malaria cases when given before the malaria season in areas with highly seasonal transmission [22–26]. However, the protective effects of RTS,S/AS01 and R21/Matrix-M are short-lived, and the population-level impact might depend on the local transmission intensity. Furthermore, there is limited data on vaccination of adults [27]. Thus, while vaccines are a potentially powerful tool in combating malaria, effective deployment strategies are needed to maximize their benefits across transmission intensity and seasonality scenarios.

Previous clinical studies investigating the efficacy of RTS,S/AS01 have focused on vaccination schedules and programs consistent with the WHO guidelines and the Malaria Vaccine Implementation Programme [25, 28]. The current recommended vaccination regime is that children aged between 5 and 17 months receive 3 doses of the vaccine spaced one month apart and then a fourth dose 18 months after dose three. Although most clinical studies do not explicitly investigate the impact of seasonality, one of the phase 3 trial sites was located in an area of Nanoro, Burkina Faso with highly seasonal transmission, providing some empirical data on efficacy in this type of setting. Moreover, seasonal vaccination strategies have already been implemented with a pair of three-year RTS,S/AS01 trials in Burkina Faso and Mali, administering the first three monthly doses and subsequent annual single dose immediately before the high-transmission season in combination with seasonal malaria chemoprevention (SMC) [29]. They find that seasonal malaria vaccination in addition to SMC is the most effective treatment regime. A recent follow-up study, in which the majority of participants re-enrolled and received two further years of seasonal RTS,S/SMC combination treatment, showed that similar protection levels could be sustained for children up to age five [30].

Several recent modeling studies quantified the impact of the RTS,S/AS01 malaria vaccine using a seasonally targeted approach. Camponovo et al. [31] used the stochastic individual-based *Open Malaria* model to investigate seasonal RTS,S/AS01 deployment in combination with SMC therapy using a seasonal transmission profile modeled after that typically observed in Senegal. They found synergistic effects with this combination treatment strategy but did not investigate how it might vary across different transmission and seasonality settings. Thompson et al. [32] used an individual-based model to evaluate the effectiveness of RTS,S/${\text{AS01}}_{\text{E}}$ with vaccination targeted to the malaria season. Their work considered seasonal settings with a single annual peak in rainfall, representing the West African seasonality, and the primary vaccines were administered to children (aged 5-17 months) three months before the transmission season with 80% coverage. They found that seasonally targeted RTS,S/${\text{AS01}}_{\text{E}}$ vaccination resulted in a more significant reduction in malaria cases and deaths compared to a purely age-based vaccination schedule (i.e., vaccination occurs when one hits a certain age) and that the effect depends on the transmission season duration and the transmission intensity. A similar individual-based model was adapted in Schmit et al. [33] to assess the cost-effectiveness of the R21/Matrix-M vaccine. The model was fitted to the vaccine efficacy data from the clinical trial in Nanoro, Burkina Faso, and simulated seasonal transmission settings using an age-based strategy and seasonal vaccination, where the primary doses were administered 3.5 months before the seasonal peak in clinical incidence. This work additionally studies the hybrid implementation, where the primary doses follow the age-based regimen, and one booster dose is given 3.5 months before the seasonal peak. In contrast to the results of Thompson et al., [32], Schmit et al. found that age-based and seasonal vaccination strategies resulted in a similar number of clinical cases and deaths averted in both seasonal and perennial settings. Other modeling studies on RTS,S/AS01 distribution have investigated the best way to allocate scarce vaccines between and within African countries, but did not consider the impact of seasonally targeted strategies at a sub-national level [34].

Relatively few malaria transmission models incorporated immune feedback in a way that allows straightforward assessment of its impact on disease dynamics [10, 11, 35–37]. In prior work we developed a model integrating human-mosquito interactions, immunity, and disease transmission with age-structured dynamics for humans [11]. We showed that including dynamic feedback between immunity and transmission intensity yielded realistic age-based prevalence distributions. Calibrating the model with data from sub-Saharan Africa, we conducted a preliminary study on the impact of RTS,S-type (RTS,S) vaccination under constant malaria transmission [11]. In the present work, we systematically study and determine the key parameters related to seasonal malaria transmission and immunity dynamics. To this end, we conduct a detailed global sensitivity analysis of our model. Additionally, we assess the effectiveness of seasonal vaccination strategies by examining a range of vaccination strategies with various vaccination periods and intensities under two distinct seasonality settings. These settings include a mild seasonality profile with two modes per year based on Siaya, Kenya, and a strong seasonal profile with a single peak based on Nanoro, Burkina Faso. We further compare the efficacy of these seasonal vaccinations to the more standard age-based vaccination schedule with constant year-long vaccination. In the strongly seasonal setting, implementing seasonal vaccination, starting between the peak in the mosquito population and the peak in malaria transmission, can prevent (approximately 9%) more cases per vaccine (i.e. completed vaccination course) than a constant age-based year-long approach. However, improvements in protection from seasonal vaccination strategies are more marginal under the mild seasonality setting in Siaya (approximately 1% more cases prevented per vaccine). This highlights that the decision on the deployment strategies requires a thorough analysis of the local seasonality profiles to maximize efficacy. Moreover, we assessed the additional benefits of seasonal targeting of the fourth (booster) dose of the RTS,S/AS01 vaccine following age-based implementation of the first three doses and found only marginal benefits. Our modeling framework can be used to evaluate the potential benefits and consequences of introducing seasonal vaccination strategies in regions given their specific seasonal mosquito prevalence profiles.

## 2. Model formulation

**Fig 1:**
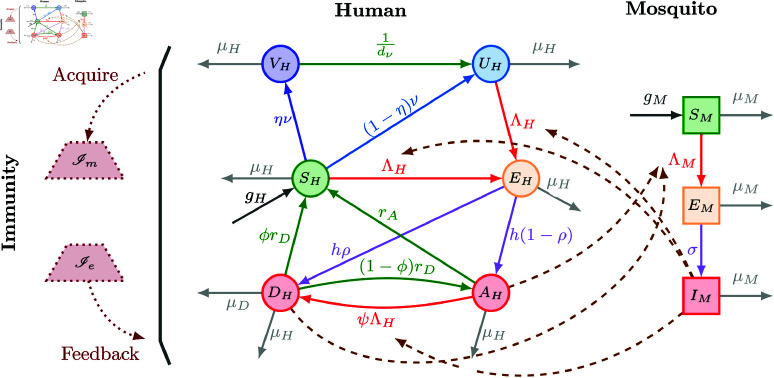
Infection dynamics flowchart for the full human-mosquito-immunity model. Solid arrows indicate the flow of individuals, dashed arrows indicate exposure that leads to infection (involving interactions between vectors and hosts), and dotted arrows represent the feedback of population immunity in humans.

Our human age-structured model combines tracking of immunity and pre-erythrocytic (targeting parasites before the blood stage) vaccination with mosquito dynamics (Fig 1). Key enhancements to the human components of the model introduced in our previous work [11] include an additional human compartment to track vaccinated but unprotected individuals (to allow the model to better reflect empirical vaccination data) and the removal of the compartment tracking of the pooled vaccine-derived immunity (as we here focus on pre-erythrocytic vaccination because the only two WHO-recommended vaccines are both pre-erythrocytic). The vaccination rate in our framework tracks the number of vaccines administered for ease of interpretation. We also remove the assumption of balanced demographics, allowing for the inclusion of natural and malaria death rates derived from empirical data. In the mosquito component, significant advancements include the relaxation of the assumption of mosquito steady-state populations by incorporating seasonal recruitment to capture observed seasonal annual entomological inoculation rate (EIR) patterns and modification of the biting rate to more realistically depend on the age of the human being bitten.

### 2.1. Human equations

The human population is stratified into six compartments related to their disease and vaccination status for *Plasmodium falciparum*: susceptible, *S*_*H*_; exposed, *E*_*H*_; asymptomatic but infectious, *A*_*H*_; symptomatic and infectious, *D*_*H*_; vaccinated and protected by sterilizing immunity, $V_{H}$; and vaccinated but unprotected against infection, *U*_*H*_. See Table 1 for state variables and key functions. The aggregate count of individuals of age-α at time *t* for α∈(0,𝙰], where 𝙰 is the finite maximal human age (100 years for numerical simulations), denoted by $P_{H}(\alpha ,t)$, is given by


$$P_{H}(\alpha ,t):=S_{H}(\alpha ,t)+V_{H}(\alpha ,t)+U_{H}(\alpha ,t)+E_{H}(\alpha ,t)+A_{H}(\alpha ,t)+D_{H}(\alpha ,t).$$

**Table 1:** Description of state variables and key functions.

Notation	Description
$S_{H}(\alpha ,t)$	Age density of susceptible humans at time *t*
$V_{H}(\alpha ,t)$	Age density of humans fully protected by vaccination at time *t*
$U_{H}(\alpha ,t)$	Age density of humans vaccinated but unprotected at time *t*
$E_{H}(\alpha ,t)$	Age density of exposed humans at time *t*
$A_{H}(\alpha ,t)$	Age density of asymptomatically infectious humans at time *t*
$D_{H}(\alpha ,t)$	Age density of symptomatically infectious humans at time *t*
$P_{H}(\alpha ,t)$	$=S_{H}+V_{H}+U_{H}+E_{H}+A_{H}+D_{H}$, age density of humans at time *t*
*N*_*H*_(*t*)	$=\int_{0}^{\text{A}}P_{H}(\alpha ,t)d\alpha$, total humans at time *t*
*S*_*M*_(*t*)	Number of susceptible mosquitoes at time *t*
*E*_*M*_(*t*)	Number of exposed mosquitoes at time *t*
*I*_*M*_(*t*)	Number of infectious mosquitoes at time *t*
*N*_*M*_(*t*)	$=S_{M}+E_{M}+I_{M}$, total mosquitoes at time *t*
$I_{e}(\alpha ,t)$	Pooled exposure-acquired immunity for all people at age α and time *t*
$I_{m}(\alpha ,t)$	Pooled maternal-derived immunity for all people at age α and time *t*
$I_{H}(\alpha ,t)$	$=c_{1}I_{e}+c_{2}I_{m}$, total pooled anti-disease immunity
${\tilde{I}}_{H}(\alpha ,t)$	$=I_{H}/P_{H}$, per-person anti-disease immunity
$\Lambda_{H}(\alpha ,t)$	Average force of infection on humans
$\Lambda_{M}(t)$	Average force of infection on mosquitoes
$f(\Lambda_{H})$	Average boosting rate of exposure-acquired immunity
$b_{H}(N_{M},N_{H},\alpha )$	Number of mosquito bites per person at age α
$b_{M}(N_{M},N_{H})$	Number of bites per mosquito

Thus, the total human population at time *t* is given by $N_{H}(t):=\int_{0}^{𝙰}P_{H}(\alpha ,t)\;d\alpha$.

Susceptible individuals, *S*_*H*_, transition to the exposed stage, *E*_*H*_, upon being bitten by an infectious mosquito, at a rate $\Lambda_{H}(\alpha ,t)$. The force of infection $\Lambda_{H}(\alpha ,t)$, found in Eq 3, depends on the biting rate, *b*_*H*_ (itself a function of age as well as mosquito and human population sizes), the infectivity of mosquitoes per infectious bite, $\beta_{M}$, and the proportion of infected mosquitoes, $I_{M}(t)/N_{M}(t)$. Upon exposure, individuals can progress directly to a symptomatic state, *D*_*H*_, with probability ρ, or remain asymptomatic, *A*_*H*_, with a probability of 1−ρ. We assume that asymptomatic individuals exhibit a lower likelihood of infecting mosquitoes than those who are symptomatic, i.e., $\beta_{A}<\beta_{D}$ [10, 38]. A fraction ϕ of symptomatic individuals recover at a rate *r*_*D*_ and transition back to the susceptible state; the remaining fraction 1−ϕ becomes asymptomatic at the same rate *r*_*D*_. Asymptomatic individuals clear parasites and subsequently recover at rate *r*_*A*_.

Infection with an additional genotype of malaria can lead to super-infection, where an individual becomes infected with multiple parasite genotypes. Although our model does not explicitly track the number of genotypes an individual harbors, it captures the potential impact of super-infection on disease progression, where asymptomatic individuals may become symptomatic due to super-infection. We assume that, with probability ψ, super-infection results in progression to symptomatic disease among the asymptomatic people, while the rest remain asymptomatic (with probability 1−ψ).

We assume only susceptible individuals can be vaccinated, which occurs following a three-dose course of the RTS,S vaccine [39] at an age-dependent rate ν(α,t). The RTS,S vaccine, with an initial efficacy after a three-dose course η, enables the individual to attain full protection against the disease upon completing the vaccination process, resulting in the population in the vaccinated and protected class, $V_{H}$. A susceptible individual remains unprotected against the disease, with probability (1−η), in which case they move to the vaccinated but unprotected state, *U*_*H*_. Individuals within the $V_{H}$ compartment also transition to *U*_*H*_ following a period of sterilizing immunity of average length $d_{\nu }$.

Individuals are born susceptible at age-dependent rate $g_{H}(\alpha )$ and die naturally at age-dependent rate $\mu_{H}(\alpha )$. See Sect 5.3 for details. People with symptomatic infection, i.e., those in compartment *D*_*H*_, can die from disease-related causes at rate $\mu_{D}$, in addition to the natural mortality rate $\mu_{H}$.

Our system of human partial differential equations (PDEs) is given by


$$\partial_{t}S_{H}+\partial_{\alpha }S_{H}=-\Lambda_{H}(\alpha ,t)S_{H}-\nu (\alpha ,t)+\phi ({\tilde{I}}_{H})r_{D}D_{H}+r_{A}A_{H}-\mu_{H}(\alpha )S_{H},$$

$$\partial_{t}V_{H}+\partial_{\alpha }V_{H}=\eta (\alpha )\;\nu (\alpha ,t)-\frac{1}{d_{\nu }}V_{H}-\mu_{H}(\alpha )V_{H},$$

$$\partial_{t}U_{H}+\partial_{\alpha }U_{H}=-\Lambda_{H}(\alpha ,t)U_{H}+\;(1-\eta (\alpha ))\;\nu (\alpha ,t)+\frac{1}{d_{\nu }}V_{H}-\mu_{H}(\alpha )U_{H},$$

$$\partial_{t}E_{H}+\partial_{\alpha }E_{H}=\Lambda_{H}(\alpha ,t)(S_{H}+U_{H})-hE_{H}-\mu_{H}(\alpha )E_{H},$$ (1)

$$\partial_{t}A_{H}+\partial_{\alpha }A_{H}=(1-\rho ({\tilde{I}}_{H}))hE_{H}-\psi ({\tilde{I}}_{H})\Lambda_{H}(\alpha ,t)A_{H}$$

$$+(1-\phi ({\tilde{I}}_{H}))r_{D}D_{H}-r_{A}A_{H}-\mu_{H}(\alpha )A_{H},$$

$$\partial_{t}D_{H}+\partial_{\alpha }D_{H}=\rho ({\tilde{I}}_{H})hE_{H}+\psi ({\tilde{I}}_{H})\Lambda_{H}(\alpha ,t)A_{H}$$

$$-r_{D}D_{H}-(\mu_{H}(\alpha )+\mu_{D}(\alpha ))D_{H},$$

with boundary conditions

$$\begin{array}{c} S_{H}(0,t)=\int_{0}^{𝙰}g_{H}(\alpha )P_{H}(\alpha ,t)\;d\alpha ,\\ V_{H}(0,t)=U_{H}(0,t)=E_{H}(0,t)=A_{H}(0,t)=D_{H}(0,t)=0, \end{array}$$ (2)

and initial conditions


$$S_{H}(\alpha ,0)=S_{H,0}(\alpha ),\;V_{H}(\alpha ,0)=V_{H,0}(\alpha ),\;U_{H}(\alpha ,0)=U_{H,0}(\alpha ),$$

$$E_{H}(\alpha ,0)=E_{H,0}(\alpha ),\;A_{H}(\alpha ,0)=A_{H,0}(\alpha ),\;D_{H}(\alpha ,0)=D_{H,0}(\alpha ),\;\alpha >0.$$

The force of infection in Eq 1 is given by

$$\Lambda_{H}(\alpha ,t)=b_{H}(N_{M}(t),N_{H}(t),\alpha )\;\beta_{M}\;\frac{I_{M}(t)}{N_{M}(t)},$$ (3)

and the biting rate function, $b_{H}(N_{M}(t),N_{H}(t),\alpha )$, is defined in Eq 4. The boundary conditions Eq 2 assume that all newborns are susceptible and do not receive the vaccination at age zero for biological realism, thus ν(0,t)=0. The description of the dynamics of the immunity level, ${\tilde{I}}_{H}$, found in Eq 1 is delayed until Sect 2.3.

### 2.2. Mosquito equations incorporating seasonality

The infection dynamics within the mosquito population advance via a system of ordinary differential equations (ODEs) tracking the states of susceptible, *S*_*M*_, exposed, *E*_*M*_, and infectious, *I*_*M*_, mosquitoes given by


$$\frac{dS_{M}}{dt}=-\Lambda_{M}(t)S_{M}+g_{M}(t)-\mu_{M}S_{M},$$

$$\frac{dE_{M}}{dt}=\Lambda_{M}(t)S_{M}-\sigma E_{M}-\mu_{M}E_{M},$$

$$\frac{dI_{M}}{dt}=\sigma E_{M}-\mu_{M}I_{M},$$

with initial conditions *S*_*M*_(0) > 0 and $E_{M}(0)=I_{M}(0)=0$. The force of infection for mosquitoes is defined as


$$\Lambda_{M}(t)=b_{M}(N_{M}(t),N_{H}(t))\frac{1}{N_{H}(t)}\int_{0}^{𝙰}(\beta_{D}D_{H}(\alpha ,t)+\beta_{A}A_{H}(\alpha ,t))\;d\alpha ,$$

and the biting rate function, $b_{M}(N_{M}(t),N_{H}(t))$, is defined in Eq 4. The total mosquito population *N*_*M*_(*t*) is given by


$$N_{M}(t):=S_{M}(t)+E_{M}(t)+I_{M}(t).$$

We incorporate seasonality through a time-dependent mosquito recruitment rate, *g*_*M*_(*t*); more details can be found in Sect 5.2. We assume mosquitoes do not recover from infection within their lifespan, so there is no recovered compartment.

To model human-mosquito contacts, we assume that the total number of bites per unit time is given by a “compromise” biting rate function [40], which depends on the ratio of human-mosquito population sizes. We further incorporate age dependency in the biting rates [10, 32, 41] to account for varying biting surface areas among humans of different ages. The total number of bites per unit of time is given by


$$b(N_{M},N_{H})=\frac{b_{m}\;b_{h}\;N_{M}\int_{0}^{\text{A}}\zeta (\alpha )P_{H}(\alpha )\;d\alpha }{b_{m}N_{M}+b_{h}\int_{0}^{\text{A}}\zeta (\alpha )P_{H}(\alpha )\;d\alpha },$$

where *b*_*m*_ and *b*_*h*_ are the number of bites a mosquito desires given a sufficient human population and the number of bites a human can tolerate, respectively. The age dependency factor, ζ(α), is given by


$$\zeta (\alpha )=1-\varepsilon e^{-\alpha /\alpha_{0}},\;\alpha \in [0,\text{A}),$$

with $\alpha_{0}=8$ years and ε=0.85 [32]. Thus, the compromised bites per mosquito per time, $b_{M}(N_{M},N_{H})$, and bites per human per time, $b_{H}(N_{M},N_{H},\alpha )$, are given by

$$\begin{array}{ccc} b_{M}(N_{M},N_{H}) & = & \frac{b_{m}\;b_{h}\;\int_{0}^{𝙰}\zeta (\alpha )P_{H}(\alpha )\;d\alpha }{b_{m}N_{M}+b_{h}\int_{0}^{𝙰}\zeta (\alpha )P_{H}(\alpha )\;d\alpha },\\ b_{H}(N_{M},N_{H},\alpha ) & = & \frac{b_{m}\;b_{h}\;N_{M}\;\zeta (\alpha )}{b_{m}N_{M}+b_{h}\int_{0}^{𝙰}\zeta (\alpha )P_{H}(\alpha )\;d\alpha }. \end{array}$$ (4)

### 2.3. Immunity equations

The development of naturally-acquired immunity to malaria is closely associated with controlling both the disease manifestation (*anti-disease immunity*) and parasite density within the body (*anti-parasite immunity*). The level of anti-disease immunity affects three aspects of the model:

ρ: probability of progression from the exposed stage to the symptomatic, infectious stage;
ϕ: probability of directly recovering from symptomatic infection; and
ψ: probability of super-infection.

We denote the pooled exposure-acquired immunity for all people at age α and time *t* by $I_{e}(\alpha ,t)$. Furthermore, maternal antibodies passed from mother to child provide temporary protection against malaria, and $I_{m}(\alpha ,t)$ is the pooled maternal-derived immunity for all people of age α at time *t*. Thus, the total anti-disease immunity is $I_{H}(\alpha ,t)=c_{1}I_{e}(\alpha ,t)+c_{2}I_{m}(\alpha ,t)$, where *c*_1_ and *c*_2_ are scaling parameters.

As individuals are exposed to malaria through interactions with infectious mosquitoes via the force of infection $\Lambda_{H}$, found in Eq 3, these interactions boost the exposure-acquired immunity of the population by a function of the force of infection, which is given by


$$f(\Lambda_{H})=\frac{\Lambda_{H}}{\gamma \;\Lambda_{H}+1},\;\gamma \geq 0.$$

This saturation function allows a maximum boosting per unit of time. The following age-structured PDEs describe the immunity dynamics of the population:


$$\partial_{t}I_{e}+\partial_{\alpha }I_{e}=f(\Lambda_{H})\left(c_{S}S_{H}+c_{E}E_{H}+c_{A}A_{H}+c_{D}D_{H}+c_{U}U_{H}\right)$$

$$-\left(\frac{1}{d_{e}}+\mu_{H}(\alpha )+\mu_{D}(\alpha )\frac{D_{H}}{P_{H}}\right)\;I_{e},$$

$$\partial_{t}I_{m}+\partial_{\alpha }I_{m}=-\left(\frac{1}{d_{m}}+\mu_{H}(\alpha )+\mu_{D}(\alpha )\frac{D_{H}}{P_{H}}\right)I_{m},$$

with boundary conditions,


$$I_{e}(0,t)=0,\;I_{m}(0,t)=m_{0}\int_{0}^{\text{A}}g_{H}(\alpha )c_{1}I_{e}(\alpha ,t)\;d\alpha ,$$

where *m*_0_ represents the fraction of maternal immunity conferred, and initial conditions, $I_{e}(\alpha ,0)=I_{e,0}(\alpha )$, $I_{m}(\alpha ,0)=I_{m,0}(\alpha )$. Weight parameters *c*_*S*_, *c*_*E*_, *c*_*A*_, *c*_*D*_, and *c*_*U*_ determine the boosting efficacy in each disease state. Exposure-acquired and maternal-derived immunity have an average period of *d*_*e*_ and *d*_*m*_ years, respectively. Furthermore, natural or disease-induced deaths reduce the pooled immunity of the population.

To describe the immunity feedback on disease transmission through the immunity-dependent probabilities ρ, ϕ, ψ, we pick a sigmoidal-shaped linking function

$$𝒮({\tilde{I}}_{H};q^{a},q^{b},s,r)=q^{a}+\frac{q^{b}-q^{a}}{1+e^{-({\tilde{I}}_{H}-s)/r}},$$ (5)

which depends on the per-person anti-disease immunity ${\tilde{I}}_{H}(\alpha ,t)=I_{H}(\alpha ,t)/P_{H}(\alpha ,t)$. We further assume that responses in the progression probabilities to symptomatic disease are similar, that is, $\phi ({\tilde{I}}_{H})=𝒮({\tilde{I}}_{H};q_{\phi }^{0},q_{\phi }^{1},s_{\phi },r_{\phi })$, $\rho ({\tilde{I}}_{H})=𝒮({\tilde{I}}_{H};q_{\rho }^{1},q_{\rho }^{0},s_{\rho },r_{\rho })$, and $\psi ({\tilde{I}}_{H})=𝒮({\tilde{I}}_{H};q_{\psi }^{1},q_{\psi }^{0},s_{\psi },r_{\psi })$. Unless otherwise noted, we have set $q_{\phi }^{0}=q_{\rho }^{0}=q_{\psi }^{0}=0.01$, and $q_{\phi }^{1}=q_{\rho }^{1}=1$ in all of our simulations. We use reduced maximum value for ψ, $q_{\psi }^{1}=0.5$, to account for the reduced contribution of new symptomatic infections from asymptomatic individuals.

### 2.4. Parameterization and numerical scheme

All codes used to generate the results and figures are openly available in the associated GitHub repository [42]. Simulations were developed in MATLAB 2022a/2023a/2023b/2024a. We follow the implicit-explicit time-stepping scheme for age-structured PDEs developed in [11], with appropriate modifications for the model studied in this paper. Initial conditions for all simulations are the disease-free equilibrium for mosquitoes, no human immunity, and 10% in *E*_*H*_, 25% in *D*_*H*_, 25% in *A*_*H*_ and the remaining 40% in *S*_*H*_ for the human population distributed by age according to the stable age distribution [11]. The parameters are summarized in Table 2, with malaria-related parameters representing *Plasmodium falciparum*. For further details on model calibration and auxiliary parameters, see Sect 5.

**Table 2:** Parameters and their values for *Plasmodium falciparum* malaria. dist. indicates there is a distribution rather than a single value. ^♯^ indicates in the absence of seasonality. - indicates the parameter is unitless.

	Description	Unit	Value	Range	Ref.
$g_{H}(\alpha )$	Per capita birth rate of humans	${\text{day}}^{-1}$	dist.		Sect 5.3.1
$\mu_{H}(\alpha )$	Per capita non-malaria human mortality rate	${\text{day}}^{-1}$	dist.		Sect 5.3.2
$\mu_{D}(\alpha )$	Per capita malaria-induced mortality rate	${\text{day}}^{-1}$	dist.		Sect 5.3.2
1/*h*	Mean incubation period in humans	day	26		[43–45]
*r*_*A*_	Recovery rate from *A*_*H*_ to *S*_*H*_	${\text{day}}^{-1}$	1/85	1/130 ∼ 1/40	[44, 45]
*r*_*D*_	Recovery rate of *D*_*H*_ to *S*_*H*_	${\text{day}}^{-1}$	1/31	1/48 ∼ 1/15	[44, 45]
*g*_*M*_(*t*)	Recruitment rate of mosquitoes	mosquitoes/day	0.5^♯^		Sect 5.2
$\mu_{M}$	Per capita natural mortality rate of mosquitoes	${\text{day}}^{-1}$	1/14	1/21.5 ∼ 1/6.7	[46, 47]
1/σ	Mean incubation period in mosquitoes	day	10	4.8 ∼ 15.4	[10, 43, 47]
*b*_*h*_	Number of mosquito bites a human tolerates	${\text{day}}^{-1}$	5		[40]
*b*_*m*_	Number of bites a mosquito desires	${\text{day}}^{-1}$	0.6		[40]
$\beta_{M}$	Infectivity of infectious mosquitoes *I*_*M*_	-	0.35	0.23 ∼ 0.74	[10, 48]
$\beta_{D}$	Infectivity of humans with symptomatic disease	-	0.2	0.13 ∼ 0.42	[10, 49]
$\beta_{A}$	Infectivity of asymptomatic humans	-	0.1	0.07 ∼ 0.21	[10, 49]
*d*_*m*_	Average period of maternal immunity ($I_{m}$)	year	0.25		[10, 50]
*d*_*e*_	Average period of exposure-acquired immunity ($I_{e}$)	year	5	3.25 ∼ 10.5	[10, 51]
γ	**Refractory period after immune boosting**	day	10	6.5 ∼ 21	[52]
ν(α,t)	Vaccination rate for primary doses	${\text{year}}^{-1}$	dist.		Sect 5.1
η(α)	Vaccine efficacy for the primary doses	-	0.72	0.47∼1	[39], S3 App.
$d_{\nu }$	Average protection period for the primary doses	year	0.53	0.25∼0.82	[39], S3 App.
$\nu_{b}(\alpha ,t)$	Vaccination rate for the booster dose	${\text{year}}^{-1}$	dist.		Sect 5.1
$\eta_{b}(\alpha )$	Vaccine efficacy for the booster dose	-	0.8	0.52∼1	[39], S3 App.
*d*_*b*_	Average protection period for the booster dose	year	2.1	1∼3.23	[39], S3 App.
**Immunity sigmoids parameters**					
$q_{\phi }^{0}$	min of ϕ	-	0.01		Assumed
$q_{\phi }^{1}$	max of ϕ	-	1		Assumed
$q_{\rho }^{0}$	min of ρ	-	0.01		Assumed
$q_{\rho }^{1}$	max of ρ	-	1		Assumed
$q_{\psi }^{0}$	min of ψ	-	0.01		Assumed
$q_{\psi }^{1}$	max of ψ	-	0.5		Assumed
$s_{\phi }$	inflection point of ϕ	-	0.44	0.28 ∼ 0.92	Sect 5.5
$r_{\phi }$	slope of ϕ	-	3.63	2.36 ∼ 7.63	Sect 5.5
$s_{\rho }$	inflection point of ρ	-	3.3	2.15 ∼ 6.94	Sect 5.5
$r_{\rho }$	slope of ρ	-	1.64	1.07 ∼ 3.45	Sect 5.5
$s_{\psi }$	inflection point of ψ	-	3.19	2.08 ∼ 6.71	Sect 5.5
$r_{\psi }$	slope of ψ	-	1.01	0.65 ∼ 2.11	Sect 5.5
**Immunity acquisition coefficients**					
*m*_0_	Fraction of maternal immunity conferred	-	1		Assumed
*c*_1_	Weight for exposure-acquired immunity	unit/people	1		Assumed
*c*_2_	Weight for maternal-derived immunity	unit/people	1		Assumed
*c*_*S*_	Weight for immunity boosting at *S*_*H*_	unit/people	0.375	0.24 ∼ 0.79	Assumed
*c*_*E*_	Weight for immunity boosting at *E*_*H*_	unit/people	0.1	0.07 ∼ 0.21	Assumed
*c*_*A*_	Weight for immunity boosting at *A*_*H*_	unit/people	0.1	0.07 ∼ 0.21	Assumed
*c*_*D*_	Weight for immunity boosting at *D*_*H*_	unit/people	0.05	0.03 ∼ 0.11	Assumed
*c*_*U*_	Weight for immunity boosting at *U*_*H*_	unit/people	0.375	0.24 ∼ 0.79	Assumed

## 3. Results

### 3.1. Seasonal malaria transmission

Before assessing the dual impacts of seasonal malaria prevalence and vaccination, we briefly illustrate the interplay between dynamic immunity and seasonality. The seasonal malaria prevalence profile is based on empirical data from Nanoro, Burkina Faso, which experiences highly seasonal malaria transmission patterns. Details on parameterization can be found in Sect 5.2.

**Fig 2:**
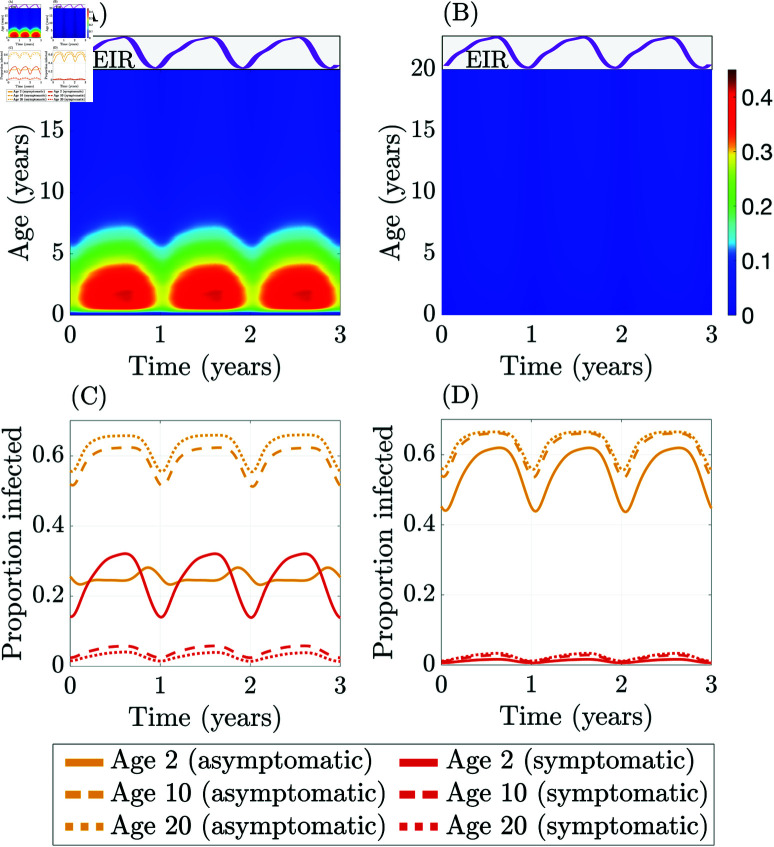
Fraction of symptomatic infections (out of total infections) under (A) dynamic immunity (population-averaged annual EIR ≈79) and (B) static high immunity where ρ≈0.03, ϕ≈0.93, ψ≈0.01 (population-averaged annual EIR ≈76). Fraction of symptomatic and asymptomatic infections (out of the total population) under (C) dynamic immunity and (D) static high immunity for age groups 2, 10, and 20 years old.

Fig 2 shows the changing level of infected individuals along the age and time dimensions for two different immunity landscapes: “dynamic” immunity (Fig 2A and 2C) and “static” high immunity (Fig 2B and 2D). Dynamic immunity involves the dynamic acquisition and waning of immunity, while for static immunity, individuals do not gain immunity from exposure or maternal antibodies; this effect is achieved by fixing the immune-dependent progression functions (ρ, ϕ, and ψ) equal to constant values to eliminate the immune feedback in the model. Under both immunity landscapes, the mosquito population is subject to seasonal forcing. Fig 2A and 2B show the fraction of symptomatic infections (out of total infections, $D_{H}/(E_{H}+A_{H}+D_{H})$), while Fig 2C and 2D show the temporal dynamics of the symptomatic and asymptomatic proportions, $D_{H}/P_{H}$ and $A_{H}/P_{H}$, within specific age cohorts. The dynamic immunity case (Fig 2A and 2C) generates an EIR, i.e., infectious bites per person per year, of approximately 79. The static immunity case (Fig 2B and 2D) generates an EIR of approximately 76; hence, these two scenarios are broadly comparable in terms of overall malaria transmission levels.

Fig 2A and 2B illustrate how the disease burden varies by age; the color bar shows the proportion of symptomatic infections as a proportion of total infections at each age. In the dynamic immunity case (Fig 2A), most symptomatic infections occur between 1 and 5 years of age, while symptomatic infections are spread more evenly between ages 1 and 20 in the static immunity case (Fig 2B). We truncate the visualization of the age dimension at 20 years, as the dynamics are essentially homogeneous in age beyond this point. Fig 2C and 2D show the proportions of symptomatic and asymptomatic infected out of the total population at each age. These plots illustrate the dramatic differences in the age structure of the disease burden for specific age cohorts under differing assumptions on immunity. For example, in the static high immunity scenario shown in Fig 2D, the two-year-old cohort (solid lines) has the lowest levels of both asymptomatic and symptomatic infection among the three cohorts; this is due to the age-dependent biting rate that generates fewer mosquito bites at lower ages owing to smaller body surface areas. Disease burden in both the asymptomatic and symptomatic compartments is increasing in age with static constant immunity. In contrast, with dynamic immune feedback, Fig 2C shows that the two-year-old cohort has by far the lowest level of asymptomatic infections among the groups considered but suffers a much higher level of symptomatic infections. Although age-dependent biting is also in force in this scenario, it is dominated by the impact of immune feedback with exposure acquired immunity in particular, allowing older cohorts to experience a significantly lower disease burden. Dynamic immunity is the most realistic and is the primary scenario studied in the remainder of the paper.

### 3.2. Sensitivity analysis

We assess the importance of various parameters on disease burden quantities to identify the key factors affecting disease transmission and mortality across different age cohorts. This analysis helps to untangle the complex interplay of malaria transmission and immunity dynamics and infer potential effective intervention strategies, especially under the seasonal transmission setting.

We begin by evaluating the sensitivity of the system in the absence of vaccination and then assess the role of vaccine-related parameters and seasonality. To do so, we perform global sensitivity analysis of the model using Latin Hypercube Sampling/Partial Rank Correlation Coefficient (LHS/PRCC) and extended Fourier Amplitude Sensitivity Test (eFAST) [53, 54]. We perform two global sensitivity analyses because LHS/PRCC provides information on the direction (positive or negative) of the association while eFAST, albeit not indicating direction, gives first-order and total-order effects. Malaria-induced deaths and malaria prevalence are the chosen quantities of interest (QOIs); see S1 Appendix for the mathematical description of these quantities. The sample size selection details are included in S2 Appendix. The QOIs are calculated at an endemic quasi-steady state among different age cohorts, including young children under 2 years (target age cohort for vaccination), children 2∼10 years (a commonly reported age range for malaria incidence studies), those above 10 years, and the entire population (if the cohort is not otherwise specified).

#### 3.2.1. Sensitivity analysis at endemic quasi-steady state

**Fig 3:**
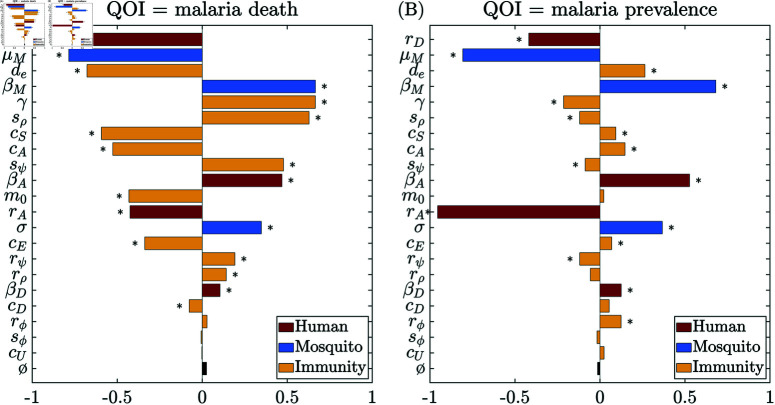
Global sensitivity analysis results using PRCC on (A) cumulative malaria death counts and (B) malaria prevalence for the entire population at endemic quasi-steady state in the absence of vaccination and seasonality. The dummy parameter (ø) is included as a null comparison. Significance (p≤0.05) is indicated with an asterisk (*). Colors indicate different categories.

**Fig 4:**
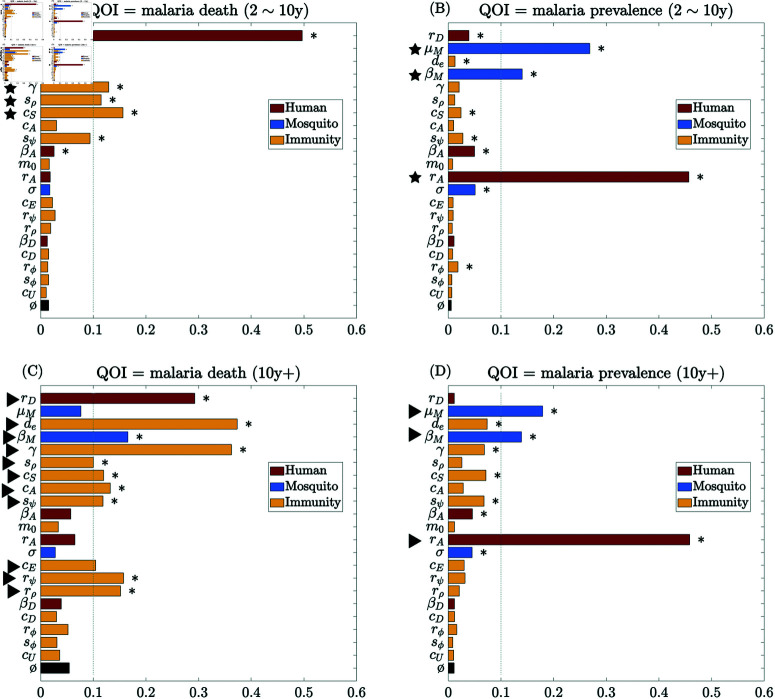
Global sensitivity analysis results using eFAST on cumulative malaria death counts (left) and malaria prevalence (right) at endemic quasi-steady state in the absence of vaccination and seasonality. (A)–(B) ages 2 ∼ 10 years; and (C)–(D) over age 10 years. Total-order sensitivity index is shown for each parameter, including a dummy parameter (ø) as a null comparison. Significance (p≤0.05) is indicated with an asterisk (*). Markers highlight the parameters with total sensitivity index above 0.1: ★=2∼10 years, ▶=10+ years, and colors indicate different categories.

Figs 3 and 4 show the global sensitivity analysis results using PRCC and eFAST on cumulative malaria death counts (left column) and prevalence (right column), at the quasi-endemic state for different age groups *in the absence of vaccination and seasonality*. The most significant parameters (from the eFAST analysis) for each age group for each QOI are summarized in Table 3.

**Table 3:** Global sensitivity analysis results in the absence of vaccination and seasonality. Markers in brackets indicate total eFAST sensitivity indices above 0.1 for the age groups: ★=2∼10 years, ▶=10+ years.

QOI	Parameters	Description
Malaria death	$r_{D}[★▶]$	recovery rate from *D*_*H*_
	$d_{e}[▶]$	average period of exposure-acquired immunity
	$\beta_{M}$ [▶]	infectivity per mosquito bite
	γ[★▶]	refractory period after immune boosting
	$s_{\rho }[★▶]$	inflection point for ρ (exposed to symptomatic probability)
	$c_{S}[★▶]$	immune boosting by susceptibles
	$c_{A}[▶]$	immune boosting by asymptomatic humans
	$s_{\psi }[▶]$	inflection point for ψ (super-infection probability)
	$c_{E}[▶]$	immune boosting by exposed humans
	$r_{\psi }[▶]$	slope for ψ (super-infection probability)
	$r_{\rho }[▶]$	slope for ρ (exposed to symptomatic probability)
Malaria prevalence	$\mu_{M}$ [★▶]	mosquito mortality rate
	$\beta_{M}$ [★▶]	infectivity per mosquito bite
	*r*_*A*_ [★▶]	recovery rate from *A*_*H*_

The PRCC sensitivity analysis for the whole population, Fig 3, shows that malaria deaths are most sensitive to the recovery rate from symptomatic infection (*r*_*D*_), and prevalence is most sensitive to the recovery rate from asymptomatic infection (*r*_*A*_). When the entire population is considered, disease-induced mortality is sensitive to many parameters spanning our model’s human, mosquito, and immune compartments. In contrast, PRCC analysis highlights relatively fewer impactful parameters concerning disease prevalence. After the asymptomatic recovery rate (*r*_*A*_), the most sensitive parameters for prevalence were both mosquito-related, namely the mosquito mortality rate ($\mu_{M}$), a proxy for mosquito population size, and the infectivity per mosquito bite ($\beta_{M}$). The recovery rate from symptomatic infection (*r*_*D*_) also plays a moderate role in disease prevalence and shows comparable sensitivity to the infectivity of asymptomatic humans ($\beta_{A}$). Overall, the PRCC population-level analysis emphasizes that the immune parameters, including immunity acquisition coefficients (*c*_*S*_, *c*_*E*_ *c*_*A*_, *c*_*D*_), refractory period of immune-boosting (γ), and the average period of immune waning (*d*_*e*_), play a more secondary role in determining disease prevalence.

Among human and immunity-related parameters, eFAST sensitivity analysis shows that malaria-induced mortality in younger children (2∼10 years) is most sensitive to the recovery rate from symptomatic infection (*r*_*D*_) (Fig 4A). Other moderately influential factors include the immune-boosting coefficient for susceptibles (*c*_*S*_), the refractory period after immune boosting (γ), and the inflection point for the symptomatic-infection sigmoid ρ ($s_{\rho }$). For the older age cohort (10+ years), immunity-related parameters play a more dominant role in determining malaria-induced mortality (Fig 4C). Malaria-induced death in this age cohort remains highly sensitive to the recovery rate from symptomatic infection (*r*_*D*_) but demonstrates an even greater sensitivity to the average period of the exposure-acquired immunity (*d*_*e*_) and the refractory period after immune boosting (γ). Additionally, disease-induced mortality exhibits moderate sensitivity to several other immunity-related parameters, including the slopes of the super-infection sigmoid ($r_{\psi }$) and symptomatic-infection sigmoid ($r_{\rho }$), followed by the immunity-boosting coefficients for susceptibles and asymptomatic (*c*_*S*_ and *c*_*A*_) and the infection point of super-infection sigmoid ($s_{\psi }$). This indicates the central role of acquired immunity in determining disease-induced mortality in older cohorts.

Deaths and prevalence are generally more sensitive to boosting at the susceptible stage and asymptomatic stages (*c*_*S*_ and *c*_*A*_) than boosting at the exposed and symptomatic stages (*c*_*E*_ and *c*_*D*_). Among the parameters for the sigmoid transition probability functions, the parameters for super-infection probability ($r_{\psi }$, $s_{\psi }$) have a comparable impact as the ones for symptomatic-infection probability ($r_{\rho }$, $s_{\rho }$). They both are consistently much more impactful than the ones for recovery probability ($r_{\phi }$, $s_{\phi }$), especially on malaria death among the older age cohort. This highlights the critical reinfection route from asymptomatic to symptomatic infection and its contribution to developing immunity against symptomatic infection.

In terms of the mosquito-related parameters, the eFAST analysis shows that malaria prevalence (Fig 4B and 4D) is very sensitive to changes in the mosquito parameters across all age cohorts, including the mosquito infectivity rate ($\beta_{M}$) and mosquito death rate ($\mu_{M}$). It is also highly sensitive to the recovery rate from asymptomatic infection (*r*_*A*_) across age cohorts. Interestingly, prevalence is less sensitive to human infectivity ($\beta_{D}$ and $\beta_{A}$) than mosquito infectivity ($\beta_{M}$). This is likely because the latter directly impacts the transmission to humans. At the same time, the impact of human infectivity is indirect and subject to other factors that also alter the force of infection on mosquitoes.

Furthermore, the sensitivity analysis highlights that mosquito-related parameters, and hence mosquito control methods, are essential in managing the overall disease prevalence. Applying insecticide and thus increasing the mosquito mortality rate ($\mu_{M}$) impacts malaria death significantly in all the cohorts considered. This finding is consistent with many prior modeling studies, such as [36, 55, 56] and the references therein. Timely malaria treatment for symptomatic infections (increasing *r*_*D*_) is critical for reducing death, while treating asymptomatic infections (increasing *r*_*A*_) is essential for controlling malaria prevalence.

#### 3.2.2. Sensitivity analysis for vaccine-related parameters at endemic quasi-steady state

We now examine the impact of vaccine-related parameters (see Sect 5.1 for specifics in parameterization) on the sensitivity analysis, including the total vaccination counts ($\nu_{0}$), the average period of sterilizing immunity ($d_{\nu }$), and vaccine efficacy (η). As in Sect 3.2.1 and S1 Appendix, the QOIs are examined at the endemic quasi-steady state; here, we restrict the QOI age range to children under 2 years old to match the target cohort of the RTS,S vaccine.

**Fig 5:**
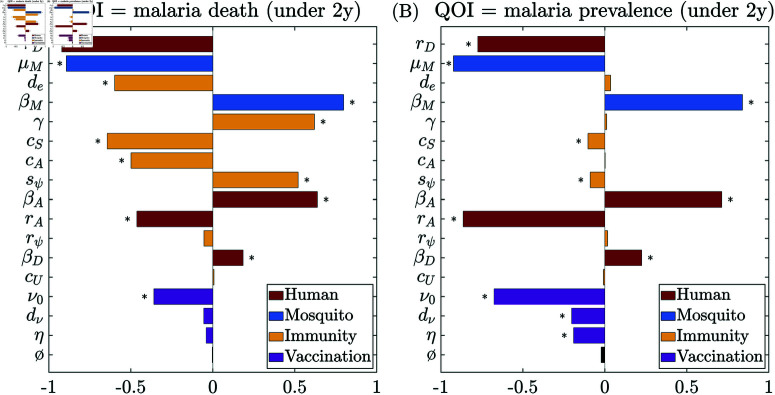
Global sensitivity analysis results using PRCC with vaccine-related parameters for children aged under 2 years for (A) cumulative malaria deaths and (B) malaria prevalence. Significance (p≤0.05) is indicated with an asterisk (*). Colors indicate different categories. See S1 Fig for the corresponding eFAST results.

Fig 5 shows the PRCC sensitivity analysis results including the aforementioned vaccination parameters (see also S1 Fig for the corresponding eFAST results). The results in S1 Fig show that the vaccination count ($\nu_{0}$) is the most impactful vaccination parameter. All three vaccination parameters have a greater impact on reducing malaria prevalence than malaria death among children under 2 years old. Despite this, the impact on malaria prevalence remains secondary to the most sensitive parameters previously determined, such as $\beta_{M}$, $\mu_{M}$, and *r*_*A*_ (Fig 5B). When considering malaria-related deaths, only the vaccination count ($\nu_{0}$) has a modest impact on the PRCC result and multiple human or mosquito-related parameters still dominate (Fig 5A). However, when running the PRCC analysis containing only the vaccination parameters (the rest of the parameters are fixed at baseline values), vaccination-related parameters indeed play a significant role in the outcomes (see S2 Fig). This is likely a result of the small age range of the cohort that the vaccine directly impacts and suggests that vaccination is secondary to other control strategies that may influence the transmission across the entire population.

#### 3.2.3. Sensitivity analysis for seasonal malaria

**Fig 6:**
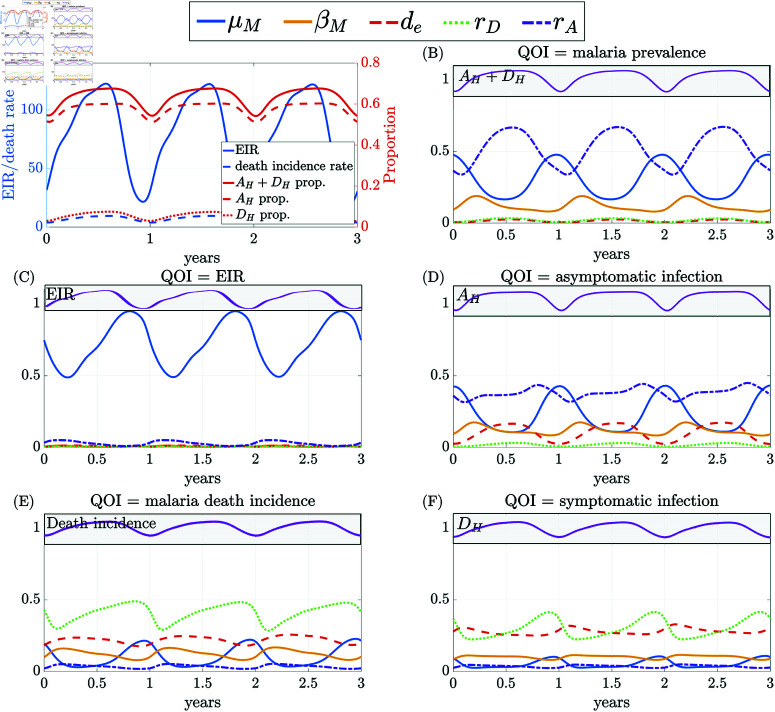
Time series of (A) quantities of interest (QOIs) and (B)–(F) total-order eFAST sensitivity indices under strong seasonal setting (Nanoro, Burkina Faso) for the entire population. The magenta curve in the gray panel shows the trend of the QOI quantity in time for (B) malaria prevalence, (C) EIR, (D) fraction asymptomatic infection, (E) malaria death incidence, and (F) fraction symptomatic infection. For readability, only selected parameters are plotted here. The eFAST results for all parameters are summarized in S3 Fig. The analogous global sensitivity analysis using PRCC is shown in S4 Fig.

We conduct global sensitivity analysis under the assumption of strong seasonality based on the Burkina Faso parameterization and track the variation of eFAST sensitivity indices versus time (Fig 6). We focus on a subset of previously determined important parameters; for complete eFAST sensitivity results, including all varied model parameters, see S3 Fig. Overall, the results under the seasonal setting identify a similar set of important parameters as in the non-seasonal setting, including the five parameters focused on in Fig 6.

The mosquito death rate ($\mu_{M}$) has a dominating impact on the EIR (Fig 6C), especially when EIR is declining. It also has a strong effect on malaria prevalence (Fig 6B) and malaria deaths (Fig 6E) with similar highly seasonal trends that peak when prevalence and fatalities are low, respectively. While more impactful than many parameters, mosquito infectivity ($\beta_{M}$) has only mild seasonal effects on all QOIs shown. The effect of mosquito parameters is consistent with the results in the non-seasonal setting, suggesting that mosquito control remains an important disease intervention for seasonal malaria control; moreover, it has a greater impact if implemented during the low-transmission season (low EIR).

The recovery rate from asymptomatic infection (*r*_*A*_) and from symptomatic infection (*r*_*D*_) also demonstrate a seasonal trend in their effects on malaria prevalence (Fig 6B). The sensitivity curves peak during the high transmission season (high EIR), suggesting that malaria treatment has a greater impact if implemented during the high transmission season. When considering different infection stages separately, *r*_*A*_ is among the most influential parameters on the fraction of asymptomatic infection but not so on symptomatic infection (Fig 6D and 6F). Similarly, *r*_*D*_ is important for symptomatic infection but not asymptomatic. Overall, the asymptomatic recovery rate, *r*_*A*_, has a much larger effect than the symptomatic recovery rate, *r*_*D*_, on the total malaria prevalence, including asymptomatic and symptomatic infections (Fig 6B), as the majority of the infections are asymptomatic in this setting (Fig 6A).

There is also a strong seasonal trend in asymptomatic human infectivity, $\beta_{A}$, with a mode shift in its impact on EIR (S3C Fig). The average period of acquired immunity (*d*_*e*_) appears to have only a modest impact on overall malaria prevalence. However, when considering the number of asymptomatic and symptomatic infections separately, the average period of acquired immunity emerges as one of the most influential parameters for both (Fig 6D and 6F). A longer average period of acquired immunity (larger *d*_*e*_) increases asymptomatic infections and decreases symptomatic infections; see S4 Fig for PRCC analysis confirming the directional effect. Other immunity-related parameters, including the boosting coefficient at the susceptible stage (*c*_*S*_), the refractory period for immunity boosting (γ), and the inflection point of the super-infection sigmoid function ($s_{\psi }$) have little influence on overall prevalence. Still, these parameters moderately impact the fraction of asymptomatic infections and disease-related deaths, especially during the peak malaria season (S3D and S3E Fig). Together with the dominating effect of *r*_*A*_ on overall malaria prevalence, these findings underscore the important role of asymptomatic infections and immune dynamics in shaping disease burden, influencing both malaria prevalence and disease-induced deaths during peak transmission periods.

### 3.3. Seasonal malaria vaccination

We assess the effectiveness of seasonal vaccination plans with different durations and start dates, assuming a fixed total number of vaccines available annually. In our model, the “start of protection” refers to the model introduction of vaccination, equivalent to when protection is conferred, assuming the completion of the first three doses. Hence, by per vaccine efficacy, we mean a full primary course of vaccination being completed. We consider two distinct seasonal transmission patterns: one with a strong seasonality profile with a single peak (based on Nanoro, Burkina Faso) and one with a mild seasonality profile with two peaks (based on Siaya, Kenya). For all simulations, we introduce vaccinations into the system at the endemic quasi-steady state and assume that each vaccination program repeats yearly and continues indefinitely. We report the vaccine efficacy for children under 2 years of age during the third year of each program to exclude the initial transient dynamics induced by vaccine introduction. We measure the vaccine efficacy by considering the cases (new symptomatic infections) prevented and deaths prevented.

**Fig 7:**
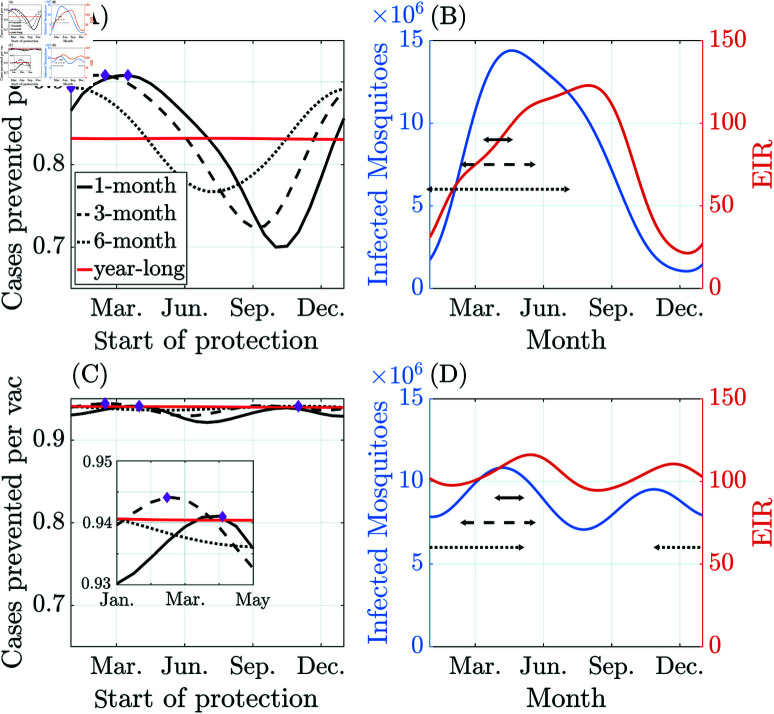
Comparison of seasonal vaccination programs in Nanoro (A)–(B) and Siaya (C)–(D) with efficacy reported for children under 2 years of age in year 3 of the program. (A, C) Cases (new symptomatic infections) are prevented per year per vaccination by programs of different durations with the 1.2 × 10^4^ total annual vaccinations fixed. The (protection) start month that maximizes the cases prevented per vac is marked with a diamond for each program. (B, D) Optimal vaccination time interval (black lines) for each seasonal vaccination campaign, dynamics of the infected mosquito populations (left y-axis in blue), and EIR (right y-axis in orange). Note: The start of protection in (A, C) refers to the introduction of vaccination, which, in our model, is equivalent to the point when protection is conferred upon completion of the first three doses.

Fig 7A compares the year-long and seasonal vaccination programs of 1-, 3-, and 6-month durations, starting at different times of the year, for the Nanoro seasonality profile. All the seasonal strategies outperform the year-long vaccination program for a wide range of starting months. The 1-month and 3-month vaccination strategies outperform the constant year-long strategy by approximately 9% in cases prevented per vaccination (the 6-month strategy outperforms the constant strategy by 7%). There are similar trends in the number of deaths prevented per vaccination in the under-two-year cohort, where seasonal strategies consistently outperform the constant year-long strategy (see S5 Fig).

For the Nanoro seasonality profile, the mosquito population (both total and infected) peaks in April (Fig 7B), and the general trend is that the longer the vaccination program lasts, the earlier the optimal starting month needs to be for that program to maximize effectiveness. Considering 1- to 3- to 6-month programs, the optimal start time of protection (i.e., the model introduction of vaccination, which is equivalent to the point when protection is conferred following the completion of the first three doses) shifts from mid-March to mid-February to the beginning of January (marked by magenta diamonds in Fig 7A). Fig 7B shows that the optimal seasonal vaccination windows for Nanoro roughly cover the peak in infected mosquitoes but precede the peak in EIR.

Using the Siaya seasonal profile, we observe only minor benefits to seasonally targeted vaccination strategies. Fig 7C shows that, although all seasonal strategies can outperform a constant vaccination campaign, the 1-, 3- and 6-month strategies only give increases in terms of cases prevented per vaccination of less than 1%. The reduced benefit of targeted strategies for Siaya owes to the mild seasonality in mosquito prevalence; Siaya’s maximal EIR amplitude is approximately 15, while Nanoro’s EIR amplitude is almost 100. However, despite the differences between these two seasonality profiles, Siaya’s optimal seasonal strategies are also concentrated just before the (highest) peak in infected mosquitoes and significantly precede the peak in EIR (Fig 7D). Moreover, regarding deaths prevented per vaccination, the seasonal strategies perform worse than the constant vaccination campaign (see S5 Fig) across the under-two-year cohort and the entire population.

The efficacy of vaccination following the initiation of a campaign can vary significantly over time in a seasonal setting. For Nanoro, the maximum efficacy of vaccination is achieved during the second year of implementation, for both the year-long vaccination and seasonal vaccination strategies. After the second year, the impact of vaccination gradually diminishes and settles at a slightly lower level due to the lower long-term EIR levels expected in a vaccinated population. The results for year three of the campaign are similar to those in year two but show less impact of the transient dynamics. We additionally tested campaigns with large enough numbers of doses to completely deplete susceptible populations but found qualitatively similar conclusions (see S6 Fig).

**Fig 8:**
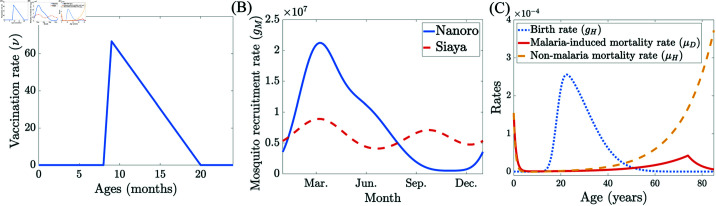
Curves from model parameterization. (A) Age-dependent vaccination rate ν(α,·) based on a triangular distribution covering the age range 8∼20 months old. (B) Daily seasonal mosquito recruitment rates *g*_*M*_(*t*) for Nanoro, Burkina Faso (solid curve) and Siaya, Kenya (dashed curve). (C) Demographic curves, including human age-dependent daily birth rate, $g_{H}(\alpha )$, malaria-induced daily mortality rate, $\mu_{D}(\alpha )$, and non-malaria-related daily mortality rate, $\mu_{H}(\alpha )$ using Kenya as a representative population.

Given that malaria vaccination programs typically include a booster dose around 24 months, we further extend our model to incorporate this additional dose (S7 Fig) and evaluate the efficacy of the booster dose under the stronger Nanoro seasonality profile. Assuming an age-based implementation of primary doses, we explore the potential advantages of the seasonal booster deployment. From S8 Fig, the booster dose prevents more cases per vaccination than the primary doses among the full population (S5B Fig), across both year-long and seasonal booster programs. However, the deaths prevented per booster dose are lower than for primary doses among the full population, which is likely due to the lower malaria-induced mortality rate among the age cohort receiving the booster dose (Fig 8C). In addition, unlike the potential seasonal advantage observed for primary doses above, seasonal booster programs generally underperform compared to the year-long program. The only exception is a limited window between January and March, where they show a marginal advantage of less than 2% in deaths prevented across the entire population.

Overall, we conclude that selecting an optimal starting time based on the duration of the vaccination campaign and the timing of the seasonality of malaria transmission is crucial for administering the primary doses. Seasonal targeting of primary doses can offer significant benefits; in Nanoro, a two-month program with protection beginning in March produces the highest number of cases prevented per vaccination (0.91), although this performance is almost identical to the 1- and 3-month campaigns shown in Fig 7A. The optimal two-month campaign prevents almost 12% more deaths per vaccination in the vaccinated age range compared to a constant vaccination program each year. However, in settings with mild seasonality, the benefits of seasonally deploying the primary doses are less clear, and more straightforward constant strategies seem to perform equivalently to more complex strategies. Additionally, the overall efficacy of seasonal booster programs, given an age-based implementation of the primary doses, can be substantially lower than that of a year-long program. These findings highlight the limitations of seasonally targeted campaigns in specific settings.

## 4. Discussion

Our age-structured PDE-ODE model accounts for the acquisition and decay of immunity resulting from exposure while incorporating vaccination effects. We include seasonality in the model by varying the mosquito recruitment rate and calibrating for two distinct transmission settings: Nanoro (Burkina Faso), which has strong seasonality, and Siaya (Kenya), which has year-round malaria transmission and mild seasonality. Our results suggest that seasonal vaccination strategies for the primary three doses of the RTS,S/AS01 vaccine can be significantly more effective (9% more cases prevented per vaccination) than a year-round vaccination program for children under 2 years, especially in regions with strong seasonality (Fig 7A). However, we did not find significant benefits to seasonal targeting of the fourth (booster) dose of RTS,S/AS01, and a purely age-based booster deployment is almost equally effective (S8 Fig). These results on the benefits of seasonal campaigns agree with those of Camponovo et al. [31] and Thompson et al. [32], which found advantages of seasonally targeted vaccination using agent-based mathematical models. The latter results are most directly comparable to ours and were based on campaigns beginning 3 months before the start of the transmission season. We tested a range of possible starting times of vaccination and found that the optimal seasonal vaccination campaign begins before the peak in infected mosquitoes for the Nanoro seasonality profile, over 5 months before the peak in the EIR (Fig 7B).

In contrast to these findings, our results for Siaya suggest that regions with flatter EIR profiles from season to season may see only marginal gains from optimally timed vaccination campaigns. Hence, seasonal targeting may not be worthwhile in settings with weaker seasonality (Fig 7C). Thus, it is not a trivial decision to implement seasonally targeted vaccination for a given region, and our modeling framework can be a valuable tool to evaluate potential vaccination strategies given local seasonality profiles.

The sensitivity analysis of our model outputs sheds light on the intricate dynamics of malaria transmission and highlights distinct patterns of parameter importance across age cohorts. Moreover, it allows us to better understand the internal structure of our complex mathematical model and identify parameters likely to significantly alter our results in different settings. Immunity-related parameters, such as the refractory period and duration of exposure-acquired immunity, immunity boosting coefficients, and sigmoid parameters, have a greater impact on disease burden in older individuals (over 10 years) than in young children (2 to 10 years old) (Fig 4). With strong seasonality in malaria transmission, there are highly seasonal trends in the sensitivity indices for the mosquito mortality rate and the immunity-related parameters (Fig 6); the mosquito mortality rate is more influential during the low-transmission season (low EIR), while the immunity-related ones are more influential in the high-transmission season (high EIR). The sensitivity analysis focusing on vaccine-related parameters provides valuable insight into their impact on malaria transmission dynamics, particularly among younger children (under 2 years). When considering variability in all parameters, the impact of vaccination parameters appears relatively limited in comparison (Fig 5), likely due to the narrow age range directly affected by the vaccine. Our analysis affirms that RTS,S vaccination alone will not be able to control or significantly curtail malaria transmission; instead, it should be used as a supplemental control strategy to reduce excess mortality in the target age ranges.

Our results are based on calibrating our model for specific locations in Kenya and Burkina Faso, so the results cannot be immediately generalized without updating the data and parameters used in calibration. However, given the appropriate demographic, mosquito prevalence, and epidemiological data, our framework and open-source codebase can readily generate similar analyses for other locations, allowing others to extend our work and investigate different scenarios. Our vaccination parameters were based on data from small vaccination trials of RTS,S/AS01 [39, 57], and we simplified the three-dose primary vaccination schedule by assuming protection starts after the third dose. Incorporating inter-dose immunity boosts could improve the accuracy of our predictions at the cost of additional complexity. Nevertheless, we do not expect these refinements to alter the qualitative conclusions of our main results. Similarly, as new vaccines with different dosing regimes and characteristics emerge, such as the R21/Matrix-M vaccine, empirical data from trials can be fed into our framework to assess their potential for seasonal targeting.

A natural extension of our work would be to consider the impact of climate change on malaria-endemic regions and the potential consequences of malaria spreading to new geographic regions, such as southern Europe and parts of the US. Several studies using statistical models have highlighted the influence of seasonal variations, likely to be amplified by climate change, on malaria cases [58, 59], and a number of recent studies have explored the effects of climate change on malaria regions and transmission [60–63]. Our framework provides a basis for studying the complex interplay between vaccination, seasonality and climate change. Further work is needed to assess climate change impacts while accounting for immune feedback, especially in scenarios when malaria emerges in regions with little to no pre-existing immunity. Such an analysis would allow an assessment of how changes in climatic factors alter not only the mosquito population but also the underlying transmission dynamics and the immune structure of the population.

Going forward, existing and emerging vaccines will be powerful tools in easing the pernicious disease burden of malaria, especially in young children. Given the expense and scarcity of these treatments, we naturally aim to optimally deploy them to areas of need, and, as our study shows, mathematical modeling can play a vital role in this targeted deployment. In particular, spatial and temporal variation in malaria transmission patterns present significant challenges and opportunities to maximize vaccination programs’ impact. Our work demonstrates that tractable and interpretable mathematical models can assess the potential merits of seasonally targeted vaccination campaigns while incorporating real-world complexities like immune feedback. Moreover, our open-source framework and codebase can be readily applied to different seasonality, vaccination, and demographic scenarios as new empirical data becomes available and seasonality patterns potentially shift due to climate change.

## 5. Methods and details of parameterization

### 5.1. Vaccine-related parameters

We follow the RTS,S/AS01 protocol in Kenya [64], where three-dose series vaccines are given to young children at 6, 7, and 9 months. A booster dose is 18 months post third dose [39]. We model the vaccination rates for the primary and booster doses, ν(α,·) and $\nu_{b}(\alpha ,\cdot )$, respectively, as triangular distributions (Fig 8A for the primary-dose distribution). The distribution for the primary doses covers the age range 8∼20 months old and has the mode at 9 months, which is the expected age upon completion of the third dose. Thus, the total vaccination count for the primary doses is given by


$$\nu_{0}(t)=\int_{0}^{\text{A}}\nu (\alpha ,t)d\alpha ,$$

and each vaccination stands for a single completion of the entire three-dose series. The age distribution for the booster dose is a shift of the primary dose distribution by 18 months. Thus, it covers the age range 26∼38 with the mode at 27 months.

For all the vaccine-related numerical studies in the main text, we simulate the distribution of vaccinations at a constant rate over a prescribed time frame, subject to the availability of the eligible population. Details on how vaccine efficacy was estimated are in S3 Appendix.

### 5.2. Seasonality-related parameters

We incorporate seasonality by varying the mosquito recruitment rate, *g*_*M*_. Adapting the formulation of seasonal profiles in [65], we set


$$g_{M}(t)=S_{0}\left(c+v(1-c){\left(\frac{1+\cos(\theta_{1})}{2}\right)}^{\kappa_{1}}+(1-v)(1-c){\left(\frac{1+\cos(\theta_{2})}{2}\right)}^{\kappa_{2}}\right),$$

where


$$\theta_{1}=2\pi \left(\frac{t+t_{0}}{365}-u_{1}\right),\;\theta_{2}=2\pi \left(\frac{t+t_{0}}{365}-u_{2}\right).$$

We incorporate a shift in time for $\theta_{1}$ and $\theta_{2}$ to account for the delay from mosquito breeding to disease transmission. The parameter values are taken from Table S6 in [65], and the time shifts are calibrated to be *t*_0_ = 100 for Siaya and *t*_0_ = 180 for Nanoro such that the peaks in the EIR curves align with empirical observations. The seasonal profiles are shown in Fig 8B, and the resulting annual EIR curves have two modes in May and November for the Siaya profile and peak around August for the Nanoro profile [65]. The calibrated model gives incidences (cases per person per year) of 3.09 and 2.62 in Siaya and Nanoro, respectively [Table 1]white2015immunogenicity. The additional seasonality parameters in *g*_*M*_ are $S_{0}=1.2\times 10^{7}\;(2.6\times 10^{7})$, v=0.393(0.55), c=0.31(0.02), $\kappa_{1}=4.08\;(6.73)$, $\kappa_{2}=3.66\;(1.68)$, $u_{1}=0.003\;(0.656)$, $u_{2}=0.456\;(0.841)$, and $t_{0}=100\;(180)$ for Siaya (Nanoro).

### 5.3. Demographics parameters

#### 5.3.1. Fertility rate

Using Kenya as a representative baseline population for demographics in sub-Saharan Africa, we employ a scaled skew normal distribution [66] for the fertility rate function (Fig 8C),


$$g_{H}(\alpha )=\frac{a_{4}}{a_{1}}\varphi (\frac{\alpha /365-a_{2}}{a_{1}})\Phi (a_{3}(\frac{\alpha /365-a_{2}}{a_{1}}))/365,$$

where φ(α) and Φ(α) are the probability density function and cumulative distribution function for the standard normal distribution, respectively. As the model does not include sex, the fertility per person is assumed to be half of the fertility per woman. We denote age in days by α and fit the model to the data in [67] to obtain coefficients *a*_1_ = 12.6, *a*_2_ = 18.2, *a*_3_ = 5.8, and *a*_4_ = 3.2.

#### 5.3.2. Mortality rates

We estimate the malaria-induced mortality rate, $\mu_{D}(\alpha )$, by using nonlinear regression to fit the following models on two separate age ranges, 0∼74 years old and >74 years old (Fig 8C):


$$\mu_{D}(\alpha )=\left\{ \begin{array}{cc} \;\left(b_{0}+b_{1}e^{-b_{2}\;\alpha /365}+b_{3}e^{b_{4}\;\alpha /365}\right)/365, & 0\leq \alpha \leq 74\times 365\\ \;(\mu_{D}^{*}/365)e^{-b_{5}\;\alpha /365}, & \alpha >74\times 365 \end{array}\right.,$$

and the data from the Institute for Health Metrics and Evaluation (IHME) on malaria prevalence and deaths in Kenya from 2019 [67]. We assume that the malaria prevalence includes symptomatic infections only and that all malaria-related deaths occur in symptomatic individuals. We estimate the age-dependent disease-induced mortality rate by dividing the average daily malaria-related death count within the year 2019 in each age group by the malaria prevalence in the corresponding age group. The fitted coefficients are *b*_0_ = 1.2 × 10^−5^, *b*_1_ = 0.06, *b*_2_ = 1.03, *b*_3_ = 1.3 × 10^−4^, *b*_4_ = 0.06, *b*_5_ = 0.18, and $\mu_{D}^{*}=\mu_{D}(74$ × 365)=0.015 for continuity of the function.

We estimate the per capita non-malaria-related mortality rate, $\mu_{H}(\alpha )$, using both the IHME and the World Health Organization’s Global Health Observatory data sets [2] (both for Kenya in 2019). We calculate deaths from all causes except malaria by subtracting malaria-related deaths from the IHME data, and we fit the remaining deaths using a similar functional form (Fig 8C),


$$\mu_{H}(\alpha )=\left(d_{0}+d_{1}e^{-d_{2}\;\alpha /365}+d_{3}e^{d_{4}\;\alpha /365}\right)/365,$$

where the fitted coefficients are $d_{0}=6\times 10^{-4}$, *d*_1_ = 0.06, *d*_2_ = 1.3, $d_{3}=2.8\times 10^{-4}$, and *d*_4_ = 0.07.

### 5.4. Malaria parameters: Infection and incubation periods

We parameterize the recovery rates, *r*_*A*_ and *r*_*D*_, by estimating the average infectious periods, 1/*r*_*A*_ and 1/*r*_*D*_, using the malaria therapy data (data courtesy of Drs. Geoffrey M. Jeffery and William E. Collins) for patients that were singly infected. Malaria therapy data is a historical data set collected in the mid-1900s where infection with malaria was used to treat neurosyphilis [68, 69], ethics discussed in [70]. We separate patients based on treatment: 145 untreated patients, i.e., not treated while positive for parasites, and 150 treated patients, i.e., treated before day 30 and while they were positive for parasites. We consider a patient infectious when the number of gametocytes exceeds the threshold 10/μL, and we consider symptomatic infection (*D*_*H*_) as those infectious with symptoms (fever as temperature above 100degF) and asymptomatic infection (*A*_*H*_) as those infectious without symptoms (no recorded fever). Not all patients have data for parasites, gametocytes, fever, and prepatent period. Each analysis was done separately, and the maximum number of patients fitting the criteria was used.

**Fig 9:**
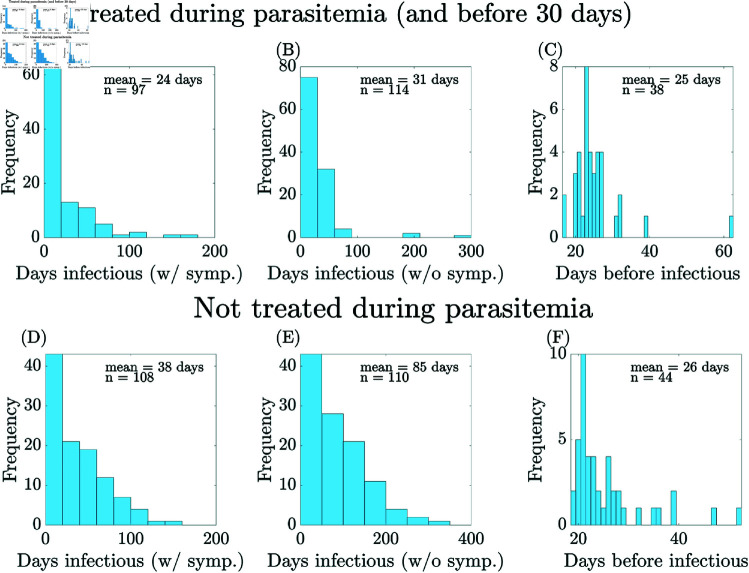
Malaria therapy data (courtesy of Drs. Jeffrey and Collins). (A–C) Patients treated while positive for parasites and before day 30 of infection. (D–F) Patients not treated while positive for parasites, i.e., never treated or treated after the end of infection. (A),(D) Length of symptomatic infection (above gametocyte threshold and showing symptoms, i.e., fever above 100degF); (B),(E) Length of asymptomatic infection (above gametocyte threshold but not showing symptoms); (C),(F) Length of period before infectiousness. See Sect 5.4 for how this data is used to calculate *r*_*D*_, *r*_*A*_, and *h*.

To estimate the symptomatic infectious period, 1/*r*_*D*_, we define the symptomatic stage (*D*_*H*_) as the period starting at the later of infectious and symptomatic and ending at the earlier of not infectious and not symptomatic. The length of these periods is shown in Fig 9A and 9D with the mean period in the *D*_*H*_ stage of 24 days (*Q*_1_ =  6 days, *Q*_3_ =  30.5 days) for treated patients and 38 days (*Q*_1_ =  9 days, *Q*_3_ =  59 days) for untreated patients. The quantities *Q*_1_ and *Q*_3_ are the first and third quartiles, which are used as initial bounds for conducting sensitivity analysis.

To estimate the fraction of people who receive effective treatment, we employed the assessment that 64% of children with fever obtained advice or treatment promptly [71]. Among children under age 5 with symptoms and who received antimalarial medication, 91% of them received artemisinin-based combination therapy (ACT). Thus, we estimate that the fraction of effective treatment is approximately 50% for children under 5 years old in the *D*_*H*_ stage. Under the assumption of a 50% effective treatment rate, this gives the mean period in the *D*_*H*_ stage as 31 days (*Q*_1_ =  7.5 days, *Q*_3_ =  45.75 days).

Similarly, we estimate the average asymptomatic infectious period, 1/*r*_*A*_. Note that there are two possible routes to *A*_*H*_: those who immediately move to asymptomatic infection ($E_{H}\to A_{H}$) or due to super-infection ($D_{H}\to A_{H}$). We consider both scenarios and define the *A*_*H*_ stage as the period starting at the time that people are infectious and show no symptoms and ending when the gametocyte level is below the defined threshold. Assuming patients do not receive treatment, as they do not show symptoms, we estimate that the mean period of the asymptomatic infectious period is 85 days (*Q*_1_ =  26 days, *Q*_3_ =  130 days) as seen in Fig 9E.

To estimate the incubation period, 1/*h*, we consider the exposed stage (*E*_*H*_) starting from the time of the infectious mosquito bite and ending on the first day that gametocytes exceed the threshold. The statistics from treated or untreated patients are very similar, and we estimate the mean incubation period as 26 days. Note that the number of patients is considerably lower here as only patients that were inoculated via mosquito bite (rather than blood transfer) were included [69].

### 5.5. Model calibration to baseline malaria transmission

We calibrate our model to the immunity curves in [10, Fig 7B], where the population’s susceptibility to developing clinical disease (corresponding to ρ in our model) is a function of age and environmental exposure levels, measured by the EIR, where $\text{EIR}=b_{H}\frac{I_{M}}{N_{M}}$. We thus calibrate our model by solving the following optimization problem numerically:


$$\min_{\Theta }\sum_{i}\sum_{j}(\rho \left(\text{EIR}(\beta_{M}^{\left(i\right)}),\alpha^{\left(j\right)};\Theta \right)-\rho^{\left(i,j\right)}{)}^{2},$$

where the vector $\Theta =\{s_{\phi },r_{\phi },s_{\rho },r_{\rho },s_{\psi },r_{\psi }\}$ contains the parameters from the sigmoid linking function 𝒮, Eq 5, which defines the immunity-dependent probabilities ρ, ϕ, ψ (see Sect 2.3 for details). We sample from different transmission scenarios by varying the mosquito-to-human infectivity parameter ($\beta_{M}^{\left(i\right)}$ between 0 and 1) and from different age groups ($\alpha^{\left(j\right)}$). Since the analytical endemic equilibrium of the system is not available, we considered the long-term solution (at time = 10 years from the initial introduction of the pathogen) and evaluated the EIR then. For a given set of sigmoid parameters, Θ, the susceptibility, ρ, is calculated from the model as a function of $\beta_{M}^{\left(i\right)}$ and $\alpha^{\left(j\right)}$. The cost function accounts for the deviation of the susceptibility value from the $\rho^{\left(i,j\right)}$ obtained from the reference curves [10] at the same EIR and age values.

**Fig 10:**
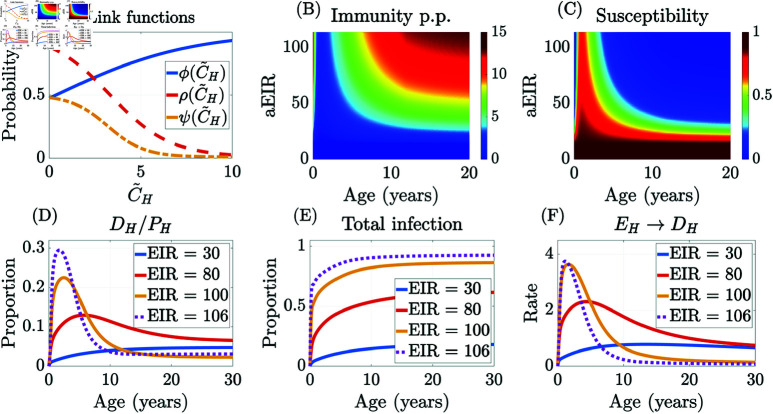
Model calibration results for sigmoid functions and population-level behavior. (A) Plots of the immune link function ϕ, ψ, and ρ as a function of the per person total immune level, ${\tilde{I}}_{H}$. (B) Heatmap showing the total immunity level per person ${\tilde{I}}_{H}$ (unitless) as a function of age and EIR. (C) Heatmap of susceptibility, $\rho ({\tilde{I}}_{H})$ (probability of progression from *E*_*H*_ to *D*_*H*_), as a function of age and EIR. (D) The proportion of symptomatic infections, $D_{H}/P_{H}$, by age and EIR. (E) Total infection, (*E*_*H*_  +  *A*_*H*_  +  $D_{H})/P_{H}$, by age and EIR. (F) The incidence rate of new symptomatic infections from the exposed stage, specifically the transition rate from $E_{H}\to D_{H}$, by age and EIR. For (D)–(F), the baseline EIR scenarios are indicated with magenta-dotted curves.

Fig 10A shows the results from the calibration process with the calibrated sigmoid functions for different progression probabilities, the heat map of immunity as a function of EIR and age in Fig 10B, and the transformed heat map of susceptibility function ρ in Fig 10C. As expected, the output in Fig 10C has very similar qualitative structure to the reference data from [10]. In the baseline scenario, without mosquito seasonality, the model exhibits a population-averaged EIR ≈106 at the quasi-endemic equilibrium (about 10 years from the initial introduction of the pathogen). Fig 10D–F show the model’s population-level predictions of symptomatic cases, overall malaria prevalence, and the rate of symptomatic incidence from exposed stage by age at different EIRs. These predictions align with field observations, where in the high-transmission regions (large EIR), the disease burden is significantly higher among young children, whereas in low-transmission regions (small EIR), the burden is more uniformly distributed across all age groups [41].

## Supporting information

S1 Appendix Formulae for quantities of interest (QOIs) for sensitivity analysis. (PDF)

S2 Appendix Selection of sample sizes for PRCC and eFAST analysis. (PDF)

S3 Appendix Patch model for vaccine calibration. Detailed description of the mathematical model of vaccination programs and how vaccine efficacy is tracked in the population.
(PDF)

S1 FigAdditional eFAST sensitivity analysis at the endemic quasi-steady state.Global sensitivity analysis (eFAST) results for vaccine-related parameters at the endemic quasi-steady state for QOI (A) malaria deaths for the age under 2 years old cohort, (B) malaria prevalence for the age under 2 years old cohort, (C) malaria death for the entire population, and (D) malaria prevalence for the entire population. Total-order sensitivity analysis indices for each parameter, including a dummy parameter (ø) as a null comparison. Significance (p≤0.05) is indicated with an asterisk (*). Colors indicate different categories. See Fig 5 for the corresponding PRCC results.(EPS)

S2 FigAdditional PRCC sensitivity analysis for vaccine-related parameters at the endemic quasi-steady state.Global sensitivity analysis results using PRCC for vaccine-related parameters at the endemic quasi-steady state for QOI (A) malaria deaths for age under 2 years old cohort and (B) malaria prevalence for age under 2 years old cohort. A dummy parameter (ø) is included as a null comparison. Significance (p≤0.05) is indicated with an asterisk (*).(EPS)

S3 Fig Additional eFAST sensitivity analysis with seasonality. Time series of (A) quantities of interest (QOIs) and (B)–(F) total-order eFAST sensitivity indices under strong seasonal setting (Burkina Faso). This is the full version, associated with Fig 6 in the main text, that includes all the parameters listed in the top panel.
(EPS)

S4 Fig Additional PRCC sensitivity analysis with seasonality. Time series of (A) quantities of interest (QOIs) and (B)-(F) PRCC indices under the strong seasonal setting (Burkina Faso). This is the PRCC version, associated with Fig 6 in the main text, which uses eFAST for selected parameters.
(EPS)

S5 FigAdditional results on seasonal vaccination for primary doses: Deaths and full population.Additional results related to Fig 7 of the main text. Comparison of seasonal vaccination programs in (A)–(B) Nanoro and (C)–(D) Siaya with efficacy reported in year 3 of the program and with the 1.2 × 10^4^ total annual vaccinations fixed. (A),(C) Deaths prevented per year per vaccination within the under-two-year cohort by programs of different durations. (B),(D) Cases prevented per year per vaccination within the entire population by programs of different durations. The (protection) start month that maximizes the deaths/cases prevented per vaccine is marked with a diamond for each program.(EPS)

S6 Fig Additional results on seasonal vaccination for primary doses: Varying number of vaccinations. Supplementary results related to Fig 7 of the main text. Seasonal vaccination strategies with the Nanoro seasonality profile compared for the under 2-year cohort in year three of the campaign (A) in terms of cases prevented per vaccination, (B) deaths prevented per vaccination, and (C) number of cases per person. Note that there are different vaccine numbers depending on the duration of the strategy. In each case, the number of vaccinations chosen is as high as possible without fully depleting the susceptible population.
(EPS)

S7 FigInfection dynamics flowchart for the modified model with a booster dose.The model includes additional compartments (denoted by primes on state variables) for populations who may be eligible for booster doses. Red-shaded boxes indicate exposure that leads to infection, contributing to either $\Lambda_{H}$ or $\Lambda_{M}$. For simplicity, immunity compartments ($I_{m}$ and $I_{e}$), human mortality rates ($\mu_{H}$ and $\mu_{D}$), and mosquito demographics (*g*_*M*_ and $\mu_{M}$) are omitted.(EPS)

S8 FigAdditional results on seasonal vaccination for booster dose.The additional efficacy of seasonal booster doses, given the age-based implementation of primary doses in Nanoro, Burkina Faso. (A),(C) Cases prevented per year per vaccination for different durations of booster deployment. (B),(D) Deaths prevented per year per vaccination for different durations of booster deployment. Efficacy is reported for year 3 of the vaccination program for children aged 2∼10 years and the entire population. The total annual vaccinations for the primary doses is 1.2 × 10^4^, with the same cap applied to annual booster doses. The x-axis represents the start of protection induced by the booster dose.(EPS)

S9 FigCalibration of vaccination parameters using data from RTS,S/AS01 vaccine Phase III clinical trial.(A) Fitting for primary doses without a booster dose. Efficacy is measured as the fractional reduction in clinical malaria incidence in the vaccinated patch ($D_{H}^{V}$), compared to the control patch ($D_{H}^{C}$). The calibration estimates for the primary doses are: initial efficacy η=0.72 and the average protection period $d_{\nu }=0.53$ years. (B) Fitting for the booster dose. Efficacy is measured as the fractional reduction in clinical malaria incidence in the vaccinated patch ($D_{H}^{V}$) and the boosted patch ($D_{H}^{B}$), compared to the control patch ($D_{H}^{C}$). The calibration estimates for the booster dose are: initial efficacy $\eta_{b}=0.8$ and the average protection period *d*_*b*_ = 2.1 years.(EPS)
