# Supplementary material for: Mathematical modeling of malaria vaccination with seasonality and immune feedback
Source: PLoS Comput Biol. 2025 May 12;21(5):e1012988. doi: 10.1371/journal.pcbi.1012988 (PMC12068631; doi:10.1371/journal.pcbi.1012988)
Supplement: S1 Appendix — (PDF) [file pcbi.1012988.s001.pdf]

S1 Appendix: Formulae for quantities of interest (QOIs) for sensitivity analysis

For sensitivity analysis at the endemic quasi-steady state, we obtain the endemic quasi-steady state by simulating the system for about 10 years after the initial introduction of the infection (10% in E_H, 25% in A_H, and 25% in D_H with the remaining 40% in S_H), then rescaled the population size to its baseline.

- QOI = malaria death (cumulative death counts) for age between α_1 and α_2 and time between t_1 and t_2:

$$M_H(\alpha, t) := \mu_D(\alpha) D_H(\alpha, t), \tag{1}$$

$$\text{QOI} = \int_{t_1}^{t_2} \int_{\alpha_1}^{\alpha_2} M_H(\alpha, t) \, \mathrm{d}\alpha \, \mathrm{d}t.$$

  For our results, we integrated the quantity over three years after the endemic quasi-steady state.

- QOI = malaria prevalence for age between α_1 and α_2:

$$\text{QOI} = \int_{\alpha_1}^{\alpha_2} A_H(\alpha, t) + D_H(\alpha, t) \, \mathrm{d}\alpha$$

- QOI = EIR (annual entomological inoculation rate) for the entire population:

$$\text{QOI} = \frac{1}{N_H} \int_0^{\mathtt{A}} b_H(\alpha, t) \frac{I_M(t)}{N_M} \times 365 \, \mathrm{d}\alpha$$

- QOI = asymptomatic infection for age between α_1 and α_2:

$$\text{QOI} = \int_{\alpha_1}^{\alpha_2} A_H(\alpha, t) \, \mathrm{d}\alpha$$

- QOI = symptomatic infection for age between α_1 and α_2:

$$\text{QOI} = \int_{\alpha_1}^{\alpha_2} D_H(\alpha, t) \, \mathrm{d}\alpha$$

- QOI = malaria death incidence for age between α_1 and α_2:

$$\text{QOI} = \int_{\alpha_1}^{\alpha_2} M_H(\alpha, t) \, \mathrm{d}\alpha = \int_{\alpha_1}^{\alpha_2} \mu_D(\alpha) D_H(\alpha, t) \, \mathrm{d}\alpha,$$

  where M_H is defined in Eq (1).
