# Supplementary material for: Mathematical modeling of malaria vaccination with seasonality and immune feedback
Source: PLoS Comput Biol. 2025 May 12;21(5):e1012988. doi: 10.1371/journal.pcbi.1012988 (PMC12068631; doi:10.1371/journal.pcbi.1012988)
Supplement: S2 Appendix — (PDF) [file pcbi.1012988.s002.pdf]

S2 Appendix: Selection of sample sizes for PRCC and eFAST analysis

For PRCC, we tested the sample sizes 100, 200, 500, and 1000, while for eFAST we set $N_S = 65, 129, 257$ and 513 (with $N_R = 5$ for resampling). We tested the adequacy of the sample size for both approaches using the top-down coefficient of concordance (TDCC), see Tables 1 and 2 below. We used a sample size of 1000 for PRCC simulations and 257 for eFAST simulations. The two approaches identify similar sensitive parameters in our study. For PRCC results, we confirmed that all QOIs fulfilled the monotonicity requirement.

Table 1. Top-Down Coefficient of Concordance (TDCC) for the PRCC. We used a sample size of 1000 for PRCC simulations. **: $p < 0.01$

	TDCC	
N	malaria death	malaria prevalence
100 vs. 200	0.94015**	0.81273**
200 vs. 500	0.94198**	0.95707**
500 vs. 1000	0.99562**	0.96745**

Table 2. Top-Down Coefficient of Concordance (TDCC) for the eFAST first-order sensitivity index ($\mathbf{S}_i$) and total-order sensitivity index ($\mathbf{S}_{T_i}$) results. We used a sample size of 257 for eFAST simulations. **: $p < 0.01$, *: $p < 0.05$

	TDCC - $\mathbf{S}_i$		TDCC - $\mathbf{S}_{T_i}$	
N	malaria death	malaria prevalence	malaria death	malaria prevalence
65 vs. 129	0.72638**	0.75802**	0.48588*	0.29376
129 vs. 257	0.95805**	0.83378**	0.56157*	0.76225**
257 vs. 513	0.99327**	0.78975**	0.55617*	0.64377**
