# Supplementary material for: Mathematical modeling of malaria vaccination with seasonality and immune feedback
Source: PLoS Comput Biol. 2025 May 12;21(5):e1012988. doi: 10.1371/journal.pcbi.1012988 (PMC12068631; doi:10.1371/journal.pcbi.1012988)
Supplement: S3 Appendix — Detailed description of the mathematical model of vaccination programs and how vaccine efficacy is tracked in the population. (PDF) [file pcbi.1012988.s003.pdf]

S3 Appendix: Patch model for vaccination calibration

To accurately determine the efficacy of vaccination, we divide the population into four groups (or patches), including (1) the vaccinated patch that consists of people who have received the primary doses of vaccination but have not yet received a booster dose, (2) the boosted patch that consists of people who received the booster dose after completing the primary doses, (3) the control patch that consists of the same number of people as the combined vaccinated and boosted patches, but who did not receive vaccination, and (4) the rest patch that includes the remaining population. Thus, the total population is given by

$$P_H(\alpha,t) = P_H^V(\alpha,t) + P_H^B(\alpha,t) + P_H^C(\alpha,t) + P_H^R(\alpha,t),$$

where P_H^V denotes the total vaccinated (not yet boosted) population, P_H^B denotes the total boosted population, P_H^C denotes the total control population, and P_H^R denotes the total rest population. Disease compartment quantities for the total population are expressed similarly, e.g., the total susceptible population is

$$S_H(\alpha,t) = S_H^V(\alpha,t) + S_H^B(\alpha,t) + S_H^C(\alpha,t) + S_H^R(\alpha,t).$$

The total human population is given by $N_H(t) = \int_0^{\mathtt{A}} P_H(\alpha,t)\,\mathrm{d}\alpha$. Patch-specific quantities involving tildes are normalized by the relevant population total for that patch, e.g., $\widetilde{D}_H^V = D_H^V/P_H^V$.

The mosquito dynamics are governed by the same equations, which are included below for readers' convenience:

$$\begin{aligned}
\frac{dS_M}{dt} &= -\Lambda_M(t)S_M + g_M - \mu_M S_M,\\
\frac{dE_M}{dt} &= \Lambda_M(t)S_M - \sigma E_M - \mu_M E_M,\\
\frac{dI_M}{dt} &= \sigma E_M - \mu_M I_M,
\end{aligned}$$

where

$$\Lambda_M(t) = b_M\big(N_M(t), N_H(t)\big)\frac{1}{N_H(t)}\int_0^{\mathtt{A}}\Big(\beta_D D_H(\alpha,t) + \beta_A A_H(\alpha,t)\Big)\,\mathrm{d}\alpha.$$

The force of infection experienced by all humans (regardless of vaccine status) is

$$\Lambda_H(t) = b_H\big(N_M(t), N_H(t)\big)\,\beta_M\,\frac{I_M(t)}{N_M(t)}.$$

We denote vaccination for the primary doses with the constant rate ν_s, efficacy η, and protection period d_ν, and booster dose with the rate ν_b, efficacy η_b, and protection period d_b. The control treatment (typically a vaccine unrelated to malaria in practice) is denoted as a constant rate ν_c. For our efficacy calibration, we assume that $\nu_s = \nu_c$, and the value of ν_b is adaptive so that all the vaccinated people receive a booster dose immediately when they are eligible. We also assume that vaccination can only be applied to susceptible populations in the rest patch. All births (and the resulting maternally conferred immunity) flow into the rest patch. In the following sections, we first give the equations for each patch (Sections 1–4), where we underline the terms that are different from the equations in the main text. We then describe the calibration details in Section 5.

1 Vaccinated patch dynamics

People move from the rest patch to the vaccinated patch due to the vaccination rate ν_s, which can be either effective $\eta\nu_s$ or not $(1-\eta)\nu_s$. Vaccinated people move to the boosted patch at a rate ν_b. The movements also lead to inflows and outflows of pooled immunity in the vaccinated patch.

The disease dynamics are given by

$$\begin{aligned}
\partial_t S_H^V + \partial_\alpha S_H^V &= -\Lambda_H(t) S_H^V + \phi(\widetilde{\mathscr{I}}_H^V) r_D D_H^V + r_A A_H^V - \mu_H(\alpha) S_H^V \underset{\sim}{-\nu_b}, \\
\partial_t V_H^V + \partial_\alpha V_H^V &= \underline{\eta\nu_s} - \frac{1}{d_\nu} V_H^V - \mu_H(\alpha) V_H^V, \\
\partial_t U_H^V + \partial_\alpha U_H^V &= -\Lambda_H(t) U_H^V \underline{+(1-\eta)\nu_s} + \frac{1}{d_\nu} V_H^V - \mu_H(\alpha) U_H^V, \\
\partial_t E_H^V + \partial_\alpha E_H^V &= \Lambda_H(t)(S_H^V + U_H^V) - h E_H^V - \mu_H(\alpha) E_H^V, \\
\partial_t A_H^V + \partial_\alpha A_H^V &= (1 - \rho(\widetilde{\mathscr{I}}_H^V)) h E_H^V - \Lambda_H(t) \psi(\widetilde{\mathscr{I}}_H^V) A_H^V + (1 - \phi(\widetilde{\mathscr{I}}_H^V)) r_D D_H^V \\
&\quad - r_A A_H^V - \mu_H(\alpha) A_H^V, \\
\partial_t D_H^V + \partial_\alpha D_H^V &= \rho(\widetilde{\mathscr{I}}_H^V) h E_H^V + \Lambda_H(t) \psi(\widetilde{\mathscr{I}}_H^V) A_H^V - r_D D_H^V - (\mu_H(\alpha) + \mu_D(\alpha)) D_H^V,
\end{aligned}$$

with the boundary conditions

$$S_H^V(0,t) = E_H^V(0,t) = A_H^V(0,t) = D_H^V(0,t) = V_H^V(0,t) = U_H^V(0,t) = 0.$$

The immune dynamics in the vaccinated patch are

$$\begin{aligned}
\partial_t \mathscr{I}_e^V + \partial_\alpha \mathscr{I}_e^V &= f(\Lambda_H) \left(c_S S_H^V + c_E E_H^V + c_A A_H^V + c_D D_H^V + c_U U_H^V \right) \\
&\quad - \left(1/d_e + \mu_H(\alpha) + \mu_D(\alpha) \widetilde{D}_H^V \right) \mathscr{I}_e^V \underline{+\nu_s \widetilde{\mathscr{I}}_e^R} \underset{\sim}{-\nu_b \widetilde{\mathscr{I}}_e^V}, \\
\partial_t \mathscr{I}_m^V + \partial_\alpha \mathscr{I}_m^V &= -\left(\frac{1}{d_m} + \mu_H(\alpha) + \mu_D(\alpha) \widetilde{D}_H^V \right) \mathscr{I}_m^V \underline{+\nu_s \widetilde{\mathscr{I}}_m^R} \underset{\sim}{-\nu_b \widetilde{\mathscr{I}}_m^V},
\end{aligned}$$

with boundary conditions,

$$\mathscr{I}_m^V(0,t) = \mathscr{I}_e^V(0,t) = 0.$$

2 Boosted patch dynamics

The disease dynamics are given by

$$\begin{aligned}
\partial_t S_H^B + \partial_\alpha S_H^B &= -\Lambda_H(t) S_H^B + \phi(\widetilde{\mathscr{I}}_H^B) r_D D_H^B + r_A A_H^B - \mu_H(\alpha) S_H^B, \\
\partial_t V_H^B + \partial_\alpha V_H^B &= \underset{\sim}{\eta_b \nu_b} - \frac{1}{d_b} V_H^B - \mu_H(\alpha) V_H^B, \\
\partial_t U_H^B + \partial_\alpha U_H^B &= -\Lambda_H(t) U_H^B + \underset{\sim}{(1-\eta_b)\nu_b} + \frac{1}{d_b} V_H^B - \mu_H(\alpha) U_H^B, \\
\partial_t E_H^B + \partial_\alpha E_H^B &= \Lambda_H(t)(S_H^B + U_H^B) - h E_H^B - \mu_H(\alpha) E_H^B, \\
\partial_t A_H^B + \partial_\alpha A_H^B &= (1 - \rho(\widetilde{\mathscr{I}}_H^B)) h E_H^B - \Lambda_H(t) \psi(\widetilde{\mathscr{I}}_H^B) A_H^B + (1 - \phi(\widetilde{\mathscr{I}}_H^B)) r_D D_H^B \\
&\quad - r_A A_H^B - \mu_H(\alpha) A_H^B, \\
\partial_t D_H^B + \partial_\alpha D_H^B &= \rho(\widetilde{\mathscr{I}}_H^B) h E_H^B + \Lambda_H(t) \psi(\widetilde{\mathscr{I}}_H^B) A_H^B - r_D D_H^B - (\mu_H(\alpha) + \mu_D(\alpha)) D_H^B,
\end{aligned}$$

with the boundary conditions

$$S_H^B(0,t) = E_H^B(0,t) = A_H^B(0,t) = D_H^B(0,t) = V_H^B(0,t) = U_H^B(0,t) = 0.$$

The immune dynamics in the vaccinated patch are

$$\begin{aligned}
\partial_t \mathscr{I}_e^B + \partial_\alpha \mathscr{I}_e^B &= f(\Lambda_H)\left(c_S S_H^B + c_E E_H^B + c_A A_H^B + c_D D_H^B + c_U U_H^B\right) \\
&\quad - \left(1/d_e + \mu_H(\alpha) + \mu_D(\alpha)\widetilde{D}_H^B\right)\mathscr{I}_e^B \underline{+\nu_b \widetilde{\mathscr{I}_e^V}}, \\
\partial_t \mathscr{I}_m^B + \partial_\alpha \mathscr{I}_m^B &= -\left(\frac{1}{d_m} + \mu_H(\alpha) + \mu_D(\alpha)\widetilde{D}_H^B\right)\mathscr{I}_m^B \underline{+\nu_b \widetilde{\mathscr{I}_m^V}},
\end{aligned}$$

with boundary conditions,

$$\mathscr{I}_m^B(0,t) = \mathscr{I}_e^B(0,t) = 0.$$

3 Control patch dynamics

In the control patch, $U_H^C(\alpha,t) = V_H^C(\alpha,t) = 0$ for all $(\alpha,t) \in \mathbb{R}_+^2$. Susceptible people move from the rest patch to the control patch at the constant rate ν_c and remain susceptible. They also bring the corresponding immunity to the control patch.

The dynamics are governed by the following equations

$$\begin{aligned}
\partial_t S_H^C + \partial_\alpha S_H^C &= -\Lambda_H(t) S_H^C + \phi(\widetilde{\mathscr{I}_H^C}) r_D D_H^C + r_A A_H^C - \mu_H(\alpha) S_H^C \underline{+\nu_c}, \\
\partial_t E_H^C + \partial_\alpha E_H^C &= \Lambda_H(t) S_H^C - h E_H^C - \mu_H(\alpha) E_H^C, \\
\partial_t A_H^C + \partial_\alpha A_H^C &= (1-\rho(\widetilde{\mathscr{I}_H^C})) h E_H^C - \Lambda_H(t)\psi(\widetilde{\mathscr{I}_H^C}) A_H^C + (1-\phi(\widetilde{\mathscr{I}_H^C})) r_D D_H^C \\
&\quad - r_A A_H^C - \mu_H(\alpha) A_H^C, \\
\partial_t D_H^C + \partial_\alpha D_H^C &= \rho(\widetilde{\mathscr{I}_H^C}) h E_H^C + \psi(\widetilde{\mathscr{I}_H^C})\Lambda_H(t) A_H^C - r_D D_H^C - (\mu_H(\alpha) + \mu_D(\alpha)) D_H^C,
\end{aligned}$$

with the boundary conditions

$$S_H^C(0,t) = E_H^C(0,t) = A_H^C(0,t) = D_H^C(0,t) = 0.$$

The immune dynamics in the control patch are

$$\begin{aligned}
\partial_t \mathscr{I}_e^C + \partial_\alpha \mathscr{I}_e^C &= f(\Lambda_H)\left(c_S S_H^C + c_E E_H^C + c_A A_H^C + c_D D_H^C\right) \underline{+\nu_c \widetilde{\mathscr{I}_e^R}} \\
&\quad - \left(1/d_e + \mu_H(\alpha) + \mu_D(\alpha)\widetilde{D}_H^C\right)\mathscr{I}_e^C, \\
\partial_t \mathscr{I}_m^C + \partial_\alpha \mathscr{I}_m^C &= -\left(\frac{1}{d_m} + \mu_H(\alpha) + \mu_D(\alpha)\widetilde{D}_H^C\right)\mathscr{I}_m^C \underline{+\nu_c \widetilde{\mathscr{I}_m^R}},
\end{aligned}$$

with boundary conditions,

$$\mathscr{I}_m^C(0,t) = \mathscr{I}_e^C(0,t) = 0.$$

4 Rest patch dynamics

In the rest patch, $U_H^R(\alpha,t) = V_H^R(\alpha,t) = 0$ for all $(\alpha,t) \in \mathbb{R}_+^2$. People move out of the rest patch due to vaccination (at rate ν_s) or due to enrollment in the control patch (at rate ν_c). Pooled immunity is also transferred to the respective patch due to the leaving population. All births (and the resulting maternally conferred immunity) flow into the rest patch.

The dynamics are governed by the following equations

$$\begin{aligned}
\partial_t S_H^R + \partial_\alpha S_H^R &= -\Lambda_H(t) S_H^R + \phi(\widetilde{\mathscr{I}}_H^R) r_D D_H^R + r_A A_H^R - \mu_H(\alpha) S_H^R \underline{- \nu_c - \nu_s},\\
\partial_t E_H^R + \partial_\alpha E_H^R &= \Lambda_H(t) S_H^R - h E_H^R - \mu_H(\alpha) E_H^R,\\
\partial_t A_H^R + \partial_\alpha A_H^R &= (1 - \rho(\widetilde{\mathscr{I}}_H^R)) h E_H^R - \Lambda_H(t) \psi(\widetilde{\mathscr{I}}_H^R) A_H^R + (1 - \phi(\widetilde{\mathscr{I}}_H^R)) r_D D_H^R\\
&\quad - r_A A_H^R - \mu_H(\alpha) A_H^R,\\
\partial_t D_H^R + \partial_\alpha D_H^R &= \rho(\widetilde{\mathscr{I}}_H^R) h E_H^R + \psi(\widetilde{\mathscr{I}}_H^R) \Lambda_H(t) A_H^R - r_D D_H^R - (\mu_H(\alpha) + \mu_D(\alpha)) D_H^R,
\end{aligned}$$

with the boundary conditions

$$S_H^R(0,t) = \int_0^{\mathtt{A}} g_H(\alpha) P_H(\alpha,t)\ \mathrm{d}\alpha, \quad E_H^R(0,t) = A_H^R(0,t) = D_H^R(0,t) = 0.$$

The immune dynamics in the rest patch are

$$\begin{aligned}
\partial_t \mathscr{I}_e^R + \partial_\alpha \mathscr{I}_e^R &= f(\Lambda_H) \left(c_S S_H^R + c_E E_H^R + c_A A_H^R + c_D D_H^R\right) \underline{-(\nu_c + \nu_s) \widetilde{\mathscr{I}}_e^R}\\
&\quad - \left(1/d_e + \mu_H(\alpha) + \mu_D(\alpha) \widetilde{D}_H^R\right) \mathscr{I}_e^R,\\
\partial_t \mathscr{I}_m^R + \partial_\alpha \mathscr{I}_m^R &= -\left(\frac{1}{d_m} + \mu_H(\alpha) + \mu_D(\alpha) \widetilde{D}_H^R\right) \mathscr{I}_m^R \underline{-(\nu_c + \nu_s) \widetilde{\mathscr{I}}_m^R},
\end{aligned}$$

with boundary conditions,

$$\mathscr{I}_m^R(0,t) = m_0 \int_0^A g_H(\alpha) c_1 \mathscr{I}_e(\alpha,t)\ \mathrm{d}\alpha, \quad \mathscr{I}_e^R(0,t) = 0.$$

5 Calibration for vaccination efficacy

We calibrated vaccination-related parameters for both primary and booster doses using the data from RTS,S/AS01 vaccine Phase III clinical trial data at Siaya site [39, 57]. For the primary doses, we calibrated the initial vaccine efficacy of the primary doses, η, and the protection period of the primary doses, d_ν, For the booster dose, we calibrated the initial vaccine efficacy of the booster dose η_b and the protection period of the booster dose d_b.

To fit the primary dose parameters, we compared the symptomatic incidence between the vaccinated patch (D_H^V) and the control patch (D_H^C) over a two-year constant vaccination period followed by a six-month observation period without vaccination. During this calibration, the booster dose remained inactive ($\nu_b = 0$). A constrained nonlinear least square algorithm was employed, where $\eta \in [0.1, 1]$ and $d_\nu \in [0.1 \times 365, 5 \times 365]$. The resulting fitted efficacy curve is shown in S9A Fig, with the fitted values of $\eta = 0.72$ and $d_\nu = 0.53$ years.

For the booster dose parameters, we fixed the primary-dose parameters (η and d_ν) and calibrated the η_b and d_b. We measure the overall efficacy of vaccination with booster by comparing the total symptomatic incidence from both the vaccinated and boosted patches with the control patch during a two-year vaccination program. For this calibration, the primary dose vaccination rate (ν_s) was kept constant rate as in the primary dose fitting, while the booster dose vaccination rate (ν_b) was dynamically adjusted to ensure all the eligible people (those who had received the primary doses, susceptible, and within the specific age cohort) received the booster dose immediately. The program (both primary and booster doses) was terminated after two years, followed by a six-month observation period without vaccination. Using the same constrained optimization method, the fitted efficacy curve with the booster is shown in S9B Fig, with the fitted values of $\eta_b = 0.8$ and $d_b = 2.1$ years.
